# Determination of Magnetic Anisotropy Tensors in Actinide Complexes Using Torque Magnetometry: A U(IV) Case Study

**DOI:** 10.1021/jacs.5c16992

**Published:** 2025-12-18

**Authors:** Leonardo Tacconi, Victor Adebayo, Laura Chelazzi, Claude Berthon, Hélène Bolvin, Mauro Perfetti

**Affiliations:** † Department of Chemistry “Ugo Schiff”, 9300 University of Florence and INSTM Research Unit, Via della Lastruccia 3-13, 50019 Sesto Fiorentino (FI), Italy; ‡ Laboratoire de Chimie et Physique Quantiques, CNRS, Université de Toulouse, 31062 Toulouse, France; § Centro di Servizi di Cristallografia Strutturale, CRIST, 50019 Sesto Fiorentino (FI), Italy; ∥ DES, ISEC, DMRC, CEA, Université Montpellier, Bagnols-sur-Cèze 30207, France

## Abstract

In this study, we employed cantilever torque magnetometry to probe the magnetic anisotropy of a single crystal of U­(DOTA)­(H_2_O) (DOTA = 1,4,7,10-tetraazacyclododecane-1,4,7,10-tetraacetic acid), representing, to the best of our knowledge, the first application of this technique to an actinide-based molecular system. Combining cantilever torque magnetometry data with *ab initio* calculations allowed us to determine the magnetic anisotropy tensor of the uranium center. The resulting parameters enabled direct comparison with the isoelectronic lanthanide complex previously reported in the literature as well as with *ab initio* predictions for the theoretical Es^4+^ isostructural analogue. We find that the magnetic anisotropy axes of **U­(DOTA)­(H_2_O)** (5*f* ^2^) closely align with those of the Pr^3+^ (4*f* ^2^) and Dy^3+^ (4*f* ^9^) analogues and with those predicted for **Es­(DOTA)­(H_2_O)** (5*f* ^9^). The combination of CTM and electronic structure calculations was essential to describe the weakly paramagnetic, EPR-silent, non-Kramers ground state of **U­(DOTA)­(H_2_O)**, despite the added complexity of four noncollinear molecules in the unit cell. Overall, these results demonstrate that cantilever torque magnetometry provides a powerful route to characterize the magnetic anisotropy of actinide complexes and offers a valuable experimental benchmark for validating computational approaches to 5f-element magnetism.

## Graphical abstract

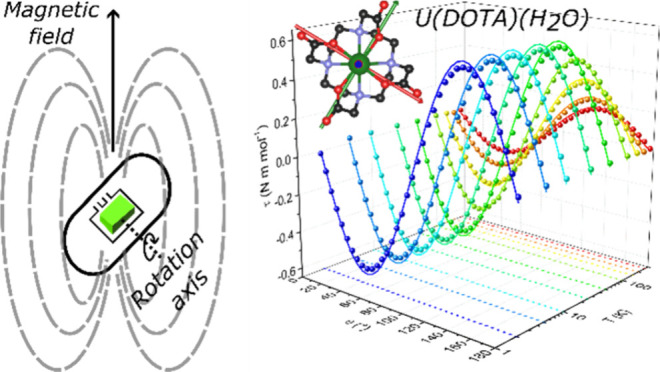

## Introduction

The study of the magnetic properties of actinide complexes is driven by fundamental questions concerning the nature of the 5*f* orbitals. Actinide complexes offer a unique platform for exploring the interplay between strong spin–orbit coupling, covalency, and low-symmetry ligand fields.^1,2^ Unlike 4*f* orbitals in lanthanide ions, 5*f* orbitals are more spatially extended and participate more actively in bonding,^3–5^ often leading to complex electronic structures.^6^ While this scenario has prompted theoretical speculation that actinides might exhibit enhanced magnetic anisotropy and improved spin dynamics, experimental realizations of competitive molecular structures encompassing actinides remain limited. For example, most reported actinide single-molecule magnets display modest blocking temperatures and rapid magnetic relaxation, with a few notable exceptions.^7–10^

However, their characterization is hindered by the challenges associated with radioactivity and the scarcity of techniques capable of probing their magnetic behavior at very low temperature. Spectroscopic measurements (magnetic circular dichroism, x-rays absorption spectroscopy, etc.) offer rich insights into the electronic structure,^11–15^ while hints on the magnetic anisotropy can be obtained exploiting electron paramagnetic resonance (EPR) selection rules,^16,17^ but these same rules can render complexes EPR silent, and temperature-induced peak broadening limits studies across wide temperature ranges. Variable temperature paramagnetic NMR can also offer valuable information,^18–20^ but it is usually not measured at cryogenic temperatures and often restricted to solution studies.^21–23^ Indeed, most magnetic characterization of actinide complexes in the solid state is limited to bulk SQUID magnetometry on powder samples,^13,24–31^ which lacks directional sensitivity and obscures anisotropic features due to averaging effects. The amount of information that can be extracted is often insufficient to provide definitive experimental insight into phenomena tied to fine details of eigenstates and eigenvalues, such as quantum tunneling between states, which strongly depends on state composition,^32,33^ or the molecular design required to achieve specific functionalities related to magnetic anisotropy.^34^ *Ab initio* calculations, although frequently employed to address these questions, still require experimental validation. Consequently, a significant gap remains in the experimental literature concerning the direct investigation of magnetic anisotropy in actinide complexes in the solid state.

In this context, Cantilever Torque Magnetometry (CTM) represents a powerful tool for molecular systems. The technique is known to deliver information about the magnetic anisotropy axes,^35^ the crystal field (or zero field splitting) parameters,^36,37^ and the thermal and field evolution of magnetic anisotropy.^38,39^ However, its usage has been so far limited to lanthanide and transition metal complexes.^40,41^ A handful of studies on actinide intermetallic compounds using CTM are present in the literature, but the technique is primarily employed to probe critical fields connected to the onset of superconductivity.^42–45^ This gap is probably due to the complexity in interpreting the torque curves, especially from systems containing noncollinear anisotropy tensors and the absence of user-friendly programs to fit the data. Nevertheless, CTM measurements do not rely on selection rules, offering a route to quasi-spectroscopic information^46^ particularly valuable for non-Kramers ions that are often EPR-silent^47,48^ and tricky to characterize by standard magnetometry alone.^49,50^

Here, we present the first CTM measurement on a molecular actinide complex, demonstrating its capability to reveal the magnetic anisotropy and even fine details of the electronic structure of the 5*f* elements. Our results show that CTM can uncover rich information about the magnetic behavior of actinide complexes, offering a valuable complement to traditional methods and paving the way for a deeper understanding of 5*f*-elements' magnetochemistry.

## Results and Discussion

### Rationale for the Choice of the Molecule

To demonstrate that CTM is a broadly applicable technique capable of addressing specific gaps in the literature, we selected a uranium-based, X-band EPR-silent molecule with a weakly magnetic ground state and a crystal packing featuring several noncollinear anisotropy tensors, conditions that are challenging to investigate using conventional single-crystal magnetometry. Among candidates meeting these criteria, the complex **U­(DOTA)­(H**_**2**_ **O)** (H_4_DOTA = 1,4,7,10-tetraazacyclododecane-1,4,7,10-tetraacetic acid) proved particularly suitable. Tetravalent uranium (5*f* ^2^) usually exhibits a well-isolated (EPR-silent) ground state.^51^ This molecule possesses a simple chemical structure (Figure 1a) but crystallizes in the orthorhombic crystal system, with several symmetry-related molecules in the unit cell. While DOTA-based complexes of lanthanides are extensively studied for their applications in magnetic resonance imaging,^52,53^ radiotherapy,^54–56^ and luminescence,^57,58^ their actinide analogues have received less attention. Nevertheless, the **Na­[Cf­(DOTA)­(H**_**2**_ **O)]** complex has been recently reported to be the first californium complex exhibiting slow relaxation of the magnetization.^59^ In the case of uranium, we noticed that there are only two publications reporting structures where U binds the unsubstituted DOTA ligand, focusing on the redox activity of the U center.^60,61^

**Figure 1:**
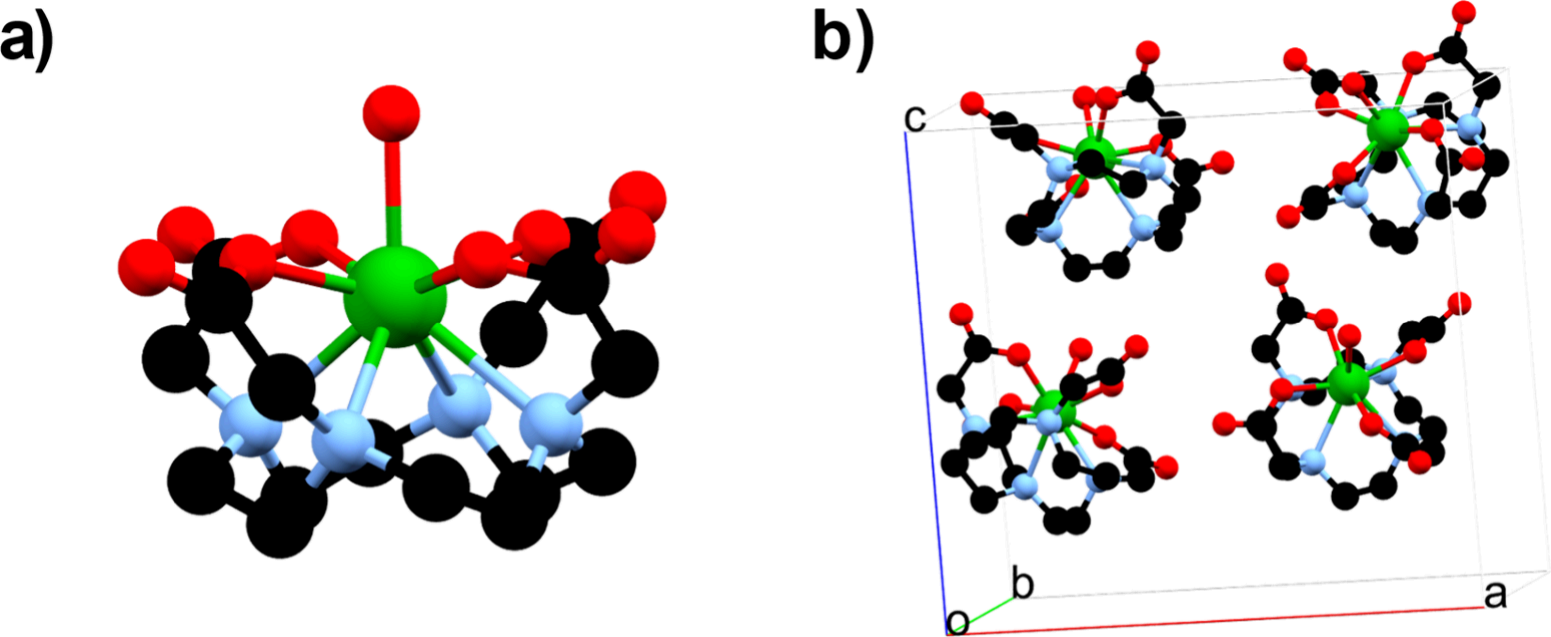
(a) Molecular structure of **U­(DOTA)­(H**_**2**_ **O)**. (b) Unit cell of the complex showing the four symmetry-related **U­(DOTA)­(H**_**2**_ **O)** molecules. Color code: uranium (green), oxygen (red), nitrogen (pale blue), and carbon (black). Hydrogen atoms and lattice water molecules have been omitted for clarity.

### Crystal Packing and Molecular Structure

The compound has been synthesized as reported in the Experimental Section (see the SI for further details). A single-crystal X-ray diffraction (SCXRD) experiment on a clear green, block-shaped single crystal revealed that **U­(DOTA)­(H**_**2**_ **O)** crystallizes in the orthorhombic space group *Pca2*_1_ (*n*. 29), with *Z* = 4 (Figure 1b). All of the crystallographic data and refinement details are summarized in Table S1. Notably, no proper symmetry elements pass through the uranium centers, indicating a C1 molecular point group symmetry for the complex. The uranium center is coordinated by the DOTA ligand in a κ^8^ binding mode, occupying the central cavity of the macrocycle and binding through four nitrogen atoms and four oxygen atoms. The coordination sphere is completed by an axial water molecule, resembling the coordination environment found in trivalent lanthanide DOTA complexes.^35^ However, unlike the tripositive lanthanide analogues, **U­(DOTA)­(H**_**2**_ **O)** is overall neutral due to the +4 oxidation state of the uranium center. Accordingly, no counterions are present in the crystal structure, only water molecules. An analysis of the uranium coordination environment reveals a close resemblance to a spherical capped squared antiprism, with a dihedral angle of 39.13° between the donor atom planes defined by the oxygen and nitrogen atoms. Within the unit cell, the four molecules (see Figure 1b), related by crystallographic *C*_2_ axes aligned with the orthorhombic main axes (*a, b, c*). Each molecule may contribute distinctively to the overall magnetic behavior, with the total magnetic anisotropy of the complex arising as the sum of the individual molecular anisotropies. This poses a significant challenge for determining the molecular magnetic anisotropy. Additionally, low-temperature X-band EPR experiments performed on a single crystal along two different crystallographic orientations did not give any measurable signal (Figure S1), indicating the singlet nature of the ground state.

### Crystal Anisotropy

To investigate the magnetic anisotropy of **U­(DOTA)­(H**_**2**_ **O)**, we performed CTM measurements on an oriented single crystal. Information regarding sample preparation is reported in Supplementary Note 1. Due to the presence of four magnetically inequivalent molecules within the unit cell, we carried out three distinct rotations of the sample, each aligned with a principal crystallographic axis, as illustrated in Figure S2. This choice reduces the number of magnetically inequivalent molecules in each measurement to two, as the rotation along a *C*_2_ symmetry axis makes pairs of susceptibility tensor projections in the perpendicular plane equal. To better visualize this, the molecular orientations of the four molecules at the start of each rotation are also depicted in Figure S2.

The high sensitivity of the technique allowed to conduct measurements across a broad temperature (2–260 K) and magnetic field (5–9 T) range, giving access to the ground state as well as to the excited state(s) contributions to the magnetic anisotropy. The torque curves obtained at *B* = 9 T are reported in Figure 2, while all of the other temperatures and fields are presented in Figures S3–S5. The zero torque angles, always 0° and 90°, are indicative of vanishing torque when a principal crystallographic axis is oriented along the magnetic field, as imposed by symmetry. Moreover, the values of the torque are remarkably small,^40^ and the curve shape is sinusoidal, indicating that the system is always within the linear response regime (*i.e.*, the magnetization versus the magnetic field is linear^40^). This hints toward a poorly anisotropic ground state of the molecules.

**Figure 2:**
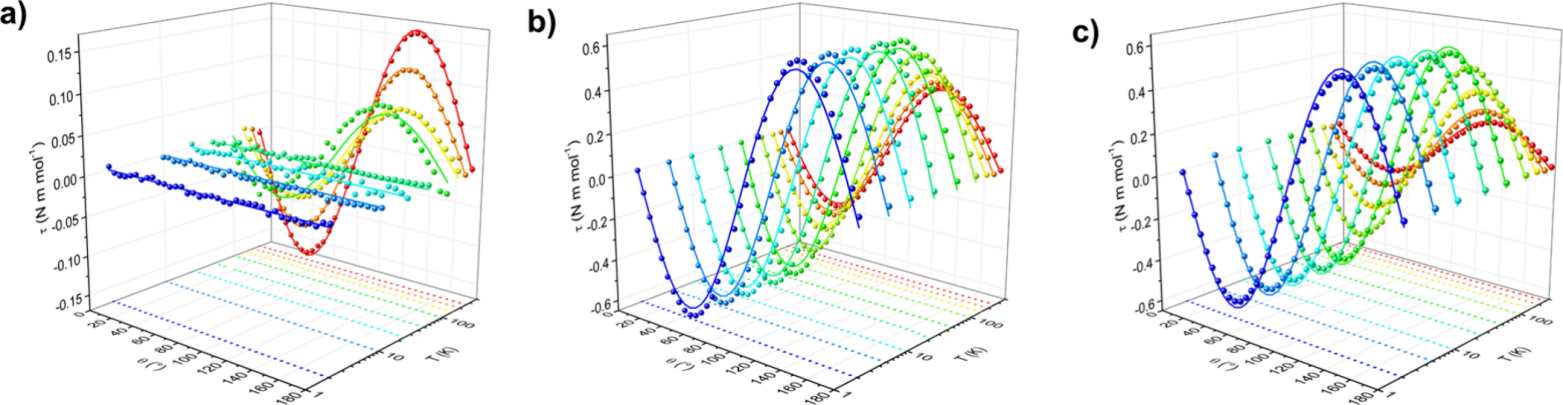
Experimental (dots) and simulated (solid lines) CTM curves acquired on **U­(DOTA)­(H**_**2**_ **O)** at 9 T and different temperatures (dashed lines) during first (panel a, along the *b* axis), second (panel b, along the *c* axis), and third (panel c, along the *a* axis) rotations. Simulations were obtained considering crystalline magnetic anisotropies, as discussed in the text.

At low temperature, no measurable signal was detected during the first rotation (Figure 2a), indicating that the crystallographic *ac* plane is nearly magnetically isotropic. In contrast, the phase of the signal obtained in the other two rotations (Figure 2b,c) identifies the *b-*axis as the hard axis of the crystalline magnetic anisotropy. Moreover, the almost equal signal intensity recorded in rotations 2 and 3 supports that the *a* and *c* crystallographic axes are magnetically equivalent. Taken together, these observations suggest that at low temperature, the **U­(DOTA)­(H**_**2**_ **O)** crystal exhibits a perfect easy-plane anisotropy, with *b* being the hard axis of the crystal.

As the temperature increases, the overall signal intensity decreases, consistent with the thermal suppression of the magnetic anisotropy. However, the temperature dependence of the signal differs significantly among the three rotations. Notably, the signal associated with the first rotation increases with the temperature (Figure 2a), indicating that the *ac* plane becomes progressively more anisotropic. At *T* = 260 K and *B* = 9 T, the signals from the first and third rotations become comparable in magnitude, although both remain smaller than the signal from the second rotation. These results suggest that at high temperatures, the magnetic anisotropy evolves such that the *a*-axis becomes the easy axis, the *b-*axis remains the hard axis, and the *c-*axis behaves as the intermediate axis. This trend can be attributed to marked rhombicity in the *ac* plane.

To extract quantitative information about the crystalline magnetic anisotropy, we performed a fit of the experimental CTM results. The magnetic torque $\vec{\tau }$ is given by the cross product of the magnetization $\vec{M}$ of the sample and applied magnetic field $\vec{B}$ , both expressed in the orthogonal crystallographic *abc* reference frame. Considering that in the linear regime $\chi =\vec{M}/\vec{B}$ , and that in our experimental setup only the component of the torque vector aligned with the rotation unitary vector ( ${\vec{r}}_{abc}$ ), denoted as τ_*r*_, can be measured, the following equation applies:

$$\tau_{r}=({\vec{M}}_{abc}\times {\vec{B}}_{abc})\cdot {\vec{r}}_{abc}=[(\chi^{\mathrm{cryst}}\cdot {\vec{B}}_{abc})\times {\vec{B}}_{abc}]\cdot {\vec{r}}_{abc}$$ 1

where **χ** ^cryst^ is the diagonal 3 × 3 crystalline magnetic susceptibility tensor expressed in the *abc* orthogonal reference frame. By defining the susceptibility anisotropies Δχ_*jk*_, as

$$\Delta \chi_{jk}=\chi_{jj}-\chi_{kk}$$ 2

with *j*, *k* ∈ {*a*, *b*, *c*}, eq 1 can be expanded in terms of tensor and vector components, leading to the following compact and general form:

$$\tau_{i}=\frac{1}{2}\sum_{j,k}\varepsilon_{ijk}\Delta \chi_{jk}B_{j}B_{k}r_{i}$$ 3

where *i*, *j*, *k* ∈ {*a*, *b*, *c*} and ε_*ijk*_ is the Levi-Civita symbol. A full derivation of eq 3 is provided in Supplementary Note 2. This equation highlights that CTM, in the linear regime, is extremely sensitive to the differences between the magnetic susceptibility components (*i.e.*, the magnetic anisotropy), rather than their absolute values. This peculiarity makes it complementary with the more widespread single-crystal magnetometry.

The simulated curves obtained by using this approach are presented in Figure 2, and the extracted anisotropy parameters are shown in Figure 3a. These results are consistent with the qualitative analysis already presented, confirming an easy-plane crystalline magnetic anisotropy, with increasing rhombicity as the temperature rises. A visual depiction of this thermal evolution is shown in Figure 3b.

**Figure 3:**
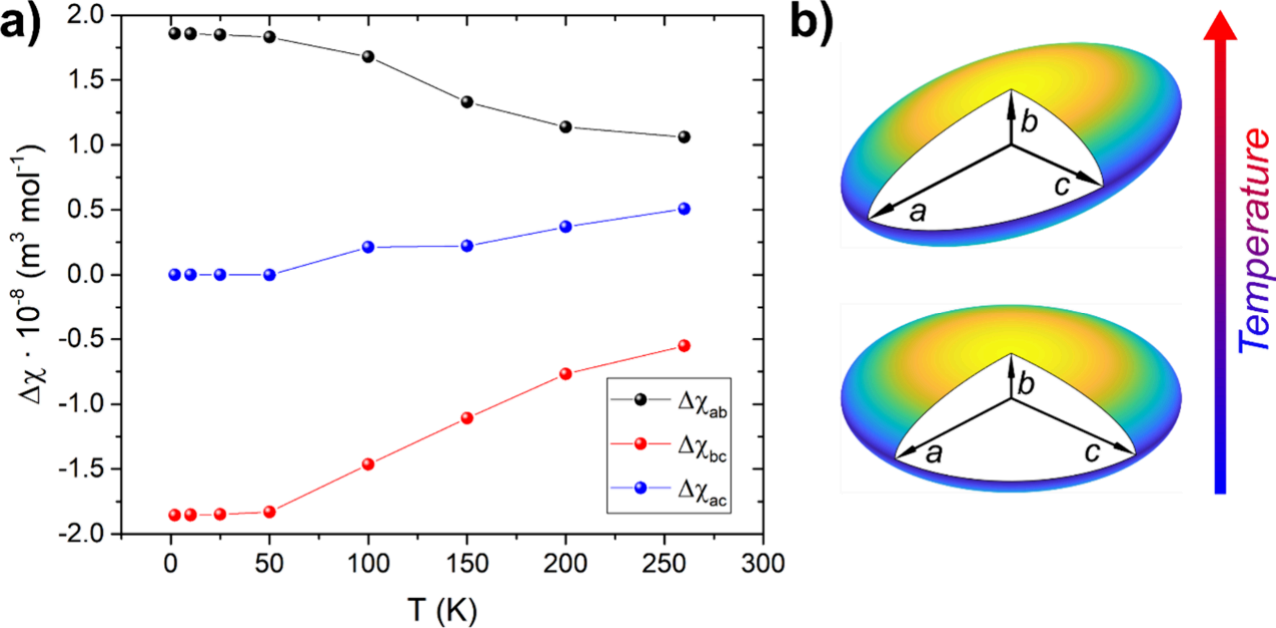
(a) Crystalline magnetic anisotropies Δχ_*ij*_ extracted from simulations of the experimental CTM measurements performed on **U­(DOTA)­(H**_**2**_ **O)**. Lines connecting the experimental points are a guide to the eye. (b) Visual representation of the thermal evolution of the crystalline magnetic susceptibility tensor. At low temperatures, the *ac* crystallographic plane is isotropic, while it becomes more rhombic at higher temperatures.

The torque measurements allowed us to unequivocally map the crystal anisotropy. However, this temperature-dependent evolution of the crystal magnetic anisotropy is caused by changes in the molecular magnetic anisotropy. Even though in many cases the torque signal can be used to directly extract single-molecule anisotropies,^37,62,63^ the present case is exceptionally complicated as it requires disentangling four contributions belonging to poorly anisotropic tensors. To gain further insight into the origin of this anisotropy and its thermal behavior and obtain a reliable starting guess for the fit of the CTM curves, we performed *ab initio* calculations.

### Molecular Anisotropy

*Ab initio* calculations (details in the Experimental Section) enabled us to predict the principal values of the susceptibility tensor of the complex and its orientation. Even though the structure does not possess any real symmetry element, our calculations predict that the hard axis lies almost exactly along the U–OH_2_ bond perpendicular to the equatorial plane defined by the DOTA ligand architecture. Such a result suggests that the crystal field can be seen as *quasi* tetragonal. This is further corroborated by the magnitude of the crystal field parameters reported in Table S2. Indeed, only the three diagonal and two off-diagonal crystal field parameters compatible with the tetragonal symmetry ( $\overset{®}{B_{4}^{4}}$ and $\overset{®}{B_{4}^{6}}$ , Wybourne formalism) possess relevant values. Further comments on the symmetry and predicted electronic structure of **U­(DOTA)­(H**_**2**_ **O)** are reported in Supplementary Note 3.

Starting from *ab initio* calculations, it is possible to simulate the experimental magnetic torque in a manner analogous to the treatment of crystalline magnetic anisotropy with an additional step. Indeed, to simulate experimental measurements, it is necessary to build the crystallographic tensor **χ** ^cryst^ as the sum of the magnetic susceptibility tensors of the four molecules in the unit cell **χ**_***i***_^mol^ (*i* = 1,2,3,4).

This transformation is accomplished using the Euler rotation matrix ***R*_*i*_**, which relates the molecular and crystallographic frames. The transformed susceptibility tensor is given by the following relation:

$$\chi^{\mathrm{cryst}}=\sum_{i=1}^{4}R_{i}^{T}\cdot \chi_{i}^{\mathrm{mol}}\cdot R_{i}$$ 4

Notice that the four Euler matrices corresponding to the four molecules of the unit cell can be derived from each other by applying symmetry operations. Specifically, the molecules are related by three distinct *C*_2_ symmetry operations along the three crystallographic axes. The Euler matrices for all four centers obtained from *ab initio* calculations are provided in Table S3. Magnetic torque is then calculated using the transformed tensors, and the total torque is obtained by summing the torque contributions from the four uranium centers.

Initially, experimental CTM curves were simulated by considering the rotation matrix and magnetic susceptibility tensors obtained from *ab initio* calculations. Results are shown in Figures S6–S14. *Ab initio* parameters lead to an overall fair agreement between the experiments and simulations with the most prominent disagreement at low temperatures. In Figures S6–S14, we also reported the contribution of each uranium center to the overall signal. As discussed before, in each rotation, we can identify two couples of uranium centers that behave identically, coherently with symmetry constraints previously discussed.

To refine the parameters extracted from *ab initio*, we decided to perform a fitting procedure of both the molecular magnetic susceptibility principal values and rotation matrices ***R*_*i*_**. More details about the fitting procedure are reported in the Experimental Section. The fitted Euler matrices for all four centers obtained from *ab initio* calculations are provided in Table S4. Using the magnetic susceptibility tensor obtained from the fitting procedure (after selecting one of the four solutions obtained from the fitting of the reference frame, see Figure S24), the agreement between experimental results and simulations becomes excellent, with simulated signals shown in Figures S15–S23. A comparison between the fitted and calculated magnetic susceptibilities is reported in Figure 4a, while Figure 4b presents the superimposed principal axes. A comparison between selected *ab initio* and fitted χ values is reported in Table S5. In both cases, the deviations from the *ab initio* values are minimal (2°, 1°, and 2° along the *x*-, *y*-, and *z*-axes, respectively), probing that the initial calculations were already remarkably accurate and validating the method.

**Figure 4:**
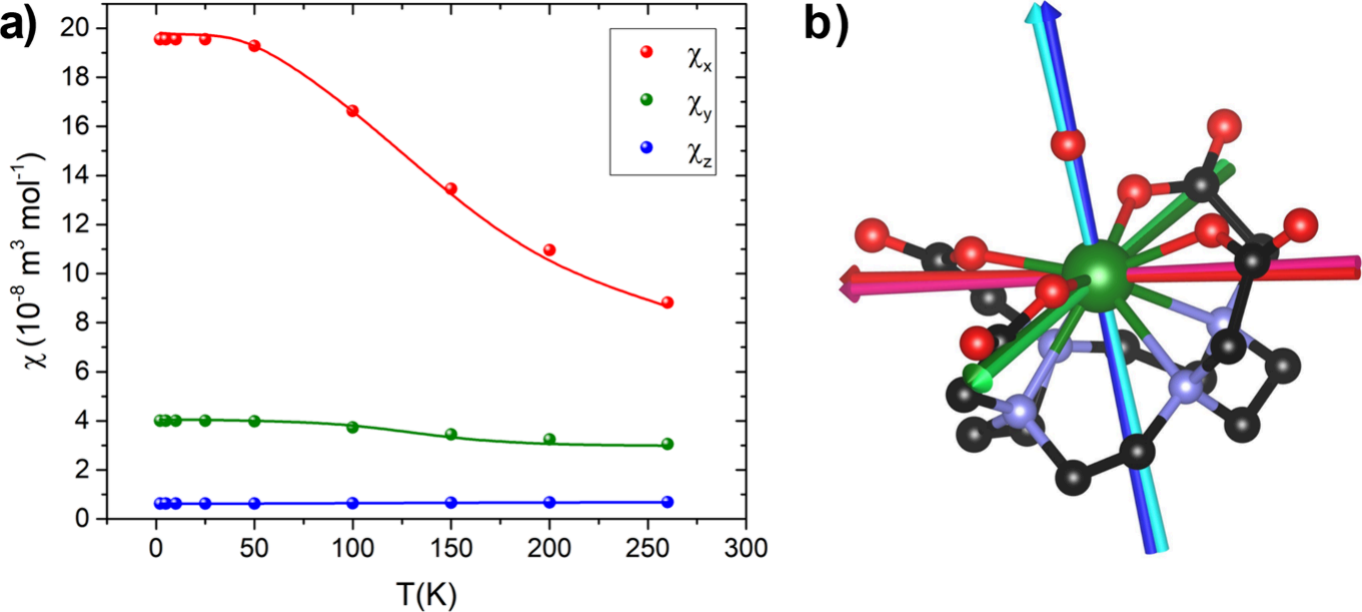
(a) Comparison between the magnetic susceptibilities obtained from *ab initio* calculations (dots) and the fitting procedure of the experimental results (solid lines), as discussed in the main text. (b) Magnetic reference frame obtained from *ab initio* calculations (x: pink, y: light green, z: cyan) and from the fitting procedure of experimental results (x: red, y: green, z: blue). Atom color scale: U, green; C, black, O: red, and N: pale blue.

The experimental validation allows for an experimentally supported discussion of the magnetic anisotropy thermal evolution. Across the entire temperature range (2–260 K), the molecular magnetic anisotropy of **U­(DOTA)­(H**_**2**_ **O)** is easy axis (*x* in Figure 4b). To emphasize the reshaping of the magnetic anisotropy, the magnetic susceptibility anisotropies were normalized with respect to the largest component, Δχ_*xz*_, and are presented in Figure 5. Notably, Δχ_*yz*_ increases with temperature, indicating an enhancement of the rhombicity within the magnetic *yz* plane, while Δχ_*xy*_ decreases. This behavior suggests that thermal effects are crucial in reshaping the magnetic anisotropy of this complex. *Ab initio* calculations provide insight into this phenomenon. Since the states are singlets (see Supplementary Note 3 and Table S6), the contribution to the magnetization uniquely arises from the coupling between states. The first excited state |1⟩ lies 169 cm^–1^ above the ground state |0⟩. The matrix element ⟨0| $\vec{M}$ |1⟩ (where $\vec{M}$ is the magnetic moment operator) lies along *x* and this coupling provides the main contribution to χ_*x*_. The coupling with the second excited state |2⟩ at 427 cm^–1^ ⟨0| $\vec{M}$ |2⟩ is instead along *y*. This is the main contribution to χ_*y*_. This contribution is smaller both because the coupling is slightly smaller (2.3 and 1.7 μ_B_, respectively) and, more importantly, because the energy gap is much larger. At 100 K, the first excited state begins to be significantly populated, contributing to reducing susceptibility along *x* (*i.e.*, reducing Δχ_*xy*_).

**Figure 5:**
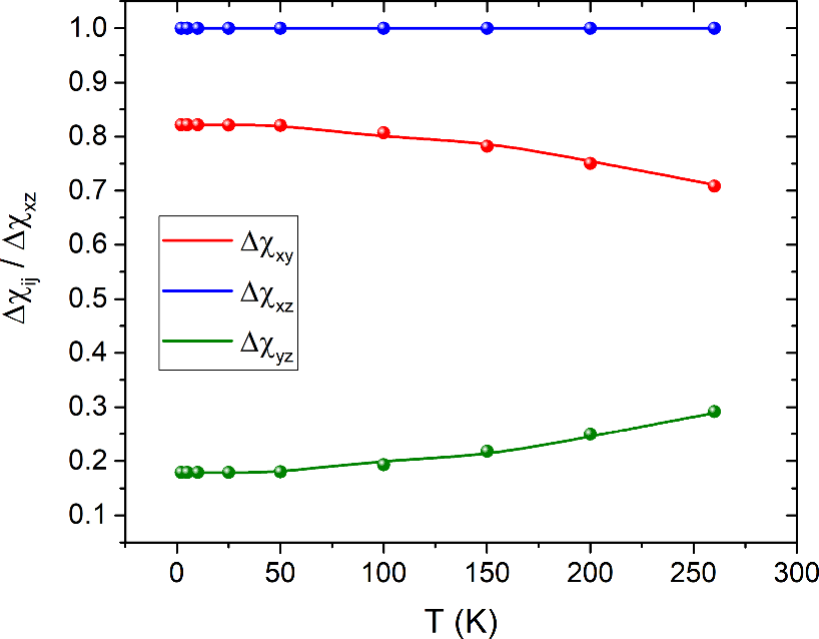
Magnetic anisotropies normalized by the Δχ_*xz*_ anisotropy obtained from *ab initio* calculations (dots) and the fitting procedure of the experimental results (solid lines). Increasing the temperature, the anisotropy Δχ_*yz*_ increases, pointing toward an increase of the rhombicity of the system.

The information extracted from the torque measurements allows a comparison of the magnetic anisotropy of our complex with the literature. Perfetti and co-workers reported the so-called *f* ^*n*+7^ effect,^35^ *i.e.*, the orientation of the magnetic anisotropy tensors of ions in isostructural lanthanide complexes differing by seven *f* electrons coincides. Such an effect was discovered on lanthanide DOTA complexes. The most immediate, purely experimental, comparison can be done between the magnetic anisotropy principal axes of **U­(DOTA)­(H**_**2**_ **O)** (5*f* ^2^) and the ones of the lanthanide-based isoelectronic structure, **Na­[Pr­(DOTA)­(H**_**2**_ **O)]** (4*f* ^2^), and of the lanthanide differing by 7*f* electrons, **Na­[Dy­(DOTA)­(H**_**2**_ **O)]** (4*f* ^9^). Despite the different complex charge, this comparison is meaningful because the coordination geometry is very similar (*e.g.*, the torsion angles between the carboxylic O and the closest N are 38.1°, 40.5°, and 38.7° for complexes containing Pr^3+^, Dy^3+^, and U^4+^, respectively). We notice that the magnetic principal axes are, also in this case, almost coincident, as visually reported in Figure 6. Interestingly, the easy axis points close to the closest M–O distance (M = Pr^3+^, Dy^3+^, or U^4+^) in all derivatives (indicated by an asterisk in all of the panels of Figure 6). Notably, this observation is independent of the degree of bond covalency, typically larger in actinides compared to lanthanides.^3,64–66^

**Figure 6:**
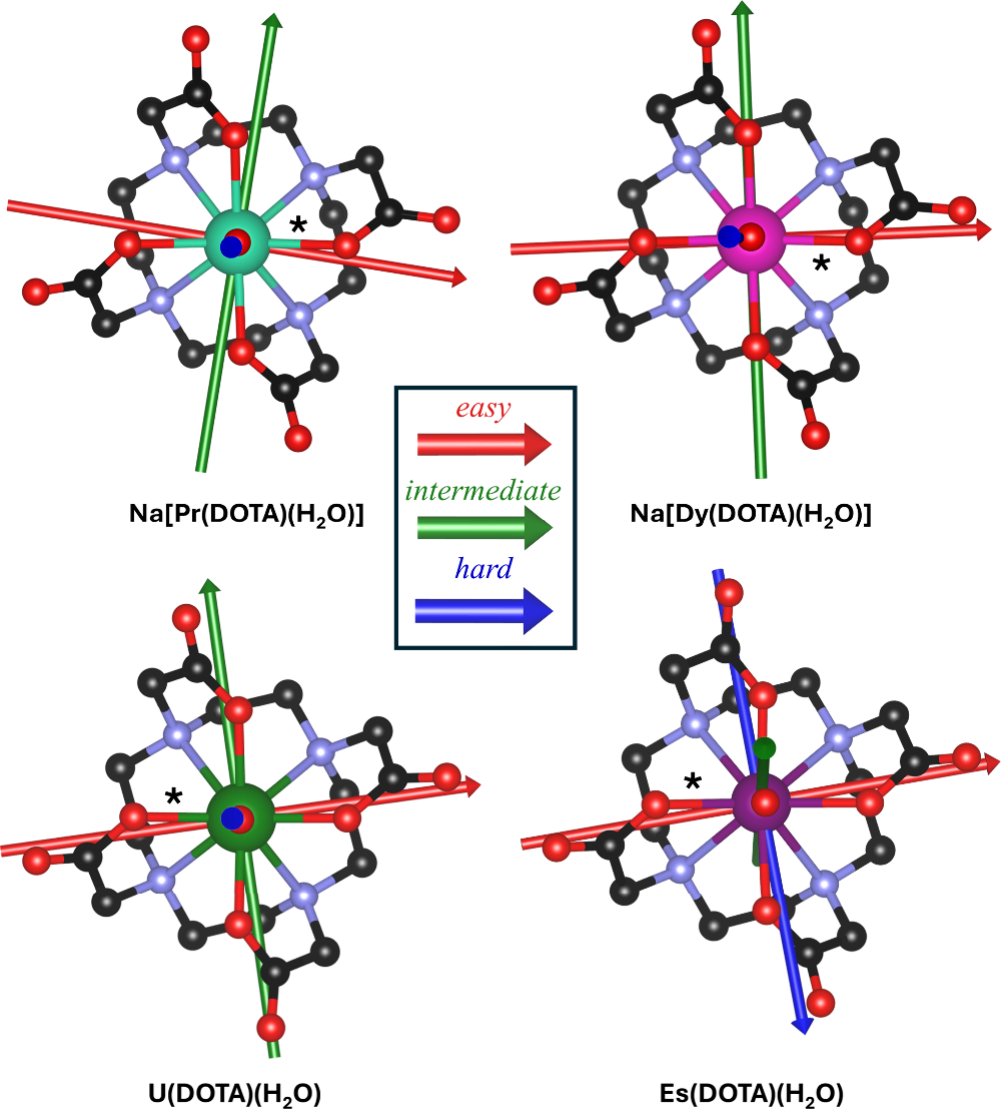
Comparison between the experimentally determined magnetic anisotropy reference frames of **Na­[Pr­(DOTA)­(H**_**2**_ **O)]**, **Na­[Dy­(DOTA)­(H**_**2**_ **O)]** (data obtained from the literature^35^), **U­(DOTA)­(H**_**2**_ **O)**, and **Es­(DOTA)­(H**_**2**_ **O)** at *T* = 2 K and *B* = 5 T. The three molecules were aligned fixing the M–O (M = metal) shortest distance (indicated by an asterisk) along the horizontal axis.

The experimental validation of the theoretical method prompted the possibility to theoretically verify the *f* ^*n*+7^ effect also in the actinide series. To this aim, we have performed *ab initio* calculations on the experimentally inaccessible Es^4+^ isostructural analogue of **U­(DOTA)­(H**_**2**_ **O)**. The structure on which the calculations were performed has been obtained by simple substitution of the metal ion inside the complex and therefore constitutes an approximation. Magnetic susceptibility tensor and energy level structures are reported in Tables S7 and S8, respectively. We find that the reference frame of **Es­(DOTA)­(H**_**2**_ **O)**, calculated at *T* = 2 K and *B* = 5 T, is closely aligned with the one of **U­(DOTA)­(H**_**2**_ **O)** (Figure 6), suggesting that the *f* ^*n*+7^ effect could extend to the actinide series. The easy axis of the structure clearly points, also in this case, along the shortest Es–O direction. The other two directions (intermediate and hard) are swapped, with respect to the other derivatives. However, we note that the hard and intermediate directions are extremely similar (*g*_hard_ = 0.04 and *g*_interm_ = 0.1), making their assignment difficult. Importantly, the anisotropy of the Es-derivative is much more pronounced compared to the U-based analogue, but this is not surprising, as observed in the lanthanide series.^35^ Finally, an interesting comparison can be made between **Es­(DOTA)­(H**_**2**_ **O)** and its lanthanide isoelectronic analogue, **Na­[Dy­(DOTA)­(H**_**2**_ **O)]**: the complexes stabilize a remarkably similar axial ground doublet (*g* = [0.04, 0.1, 19.3] and *g* = [0.255, 0.668, 19.238],^35^ respectively).

## Conclusions

The magnetic anisotropy of a single crystal of **U­(DOTA)­(H**_**2**_ **O)** was measured using CTM, applied here for the first time on an actinide complex. While the crystal magnetic anisotropy was unequivocally mapped with CTM, the synergistic combination of *ab initio* calculations and torque data in a broad range of temperatures and fields was key to describe the molecular magnetic anisotropy in detail. We therefore extracted the orientation of the anisotropy tensors, allowing us to meaningfully compare the magnetic anisotropy of our complex with the 4f^2^ (Pr^3+^) and 4f^9^ (Dy^3+^) isostructural lanthanide complexes. The magnetic anisotropy principal axes of the three complexes almost coincide. *Ab initio* calculations performed on the hypothetical Es^4+^ (5f^9^) isostructural analogue suggest that the easy axis of this complex should also coincide with the easy axis direction of the other three complexes, suggesting that the *f* ^*n*+7^ effect could be active also in the 5f series. Notably, the chosen complex was intended as a proof of concept for a particularly challenging system, namely, a 5f^2^ non-Kramers ion with a weakly magnetic ground state and four magnetically inequivalent molecules in the unit cell. Consequently, we anticipate that uranium complexes with stronger anisotropy and/or crystal structures featuring collinear tensors can be mapped with even greater accuracy. This would provide a valuable route to extract experimentally elusive information and to benchmark *ab initio* methods for actinide complexes.

## Experimental Section

### Synthesis

The complex (**UDOTA)­(H**_**2**_ **O)** was synthesized in two steps. See Supplementary Note 3 for details and the reaction scale. First, the **U­(DOTA)­(DMSO)** was prepared by complexation of UCl_4_ with DOTA­(TEA)_4_ in a dimethyl sulfoxide (DMSO) phase in a similar way to that proposed by Kent et al. in 2019.^60^ The UCl_4_ reagent was prepared from uranyl nitrate in an aqueous solution. After uranyl reduction with Rongalite (HOCH_2_SO_2_Na), U^IV^ was precipitated with NaOH, washed, and recovered with concentrated HCl. The UCl_4_ solution was dried at room temperature under a nitrogen flow. Commercial H_4_DOTA was neutralized by stirring a stoichiometric amount of triethylamine (TEA) in DMSO for 1 h, whereupon a large amount of a white compound precipitates at room temperature. This solid was added to UCl_4_ in DMSO (a slight DOTA excess was used) and heated at 60 °C for 12 h. The formation of **U­(DOTA)­(DMSO)** was monitored by ^1^H NMR spectroscopy. After completion of the reaction, the complex was precipitated by dropwise addition of this DMSO solution to a CHCl_3_ phase. The resulting powder was washed three times with THF and then dried at room temperature under nitrogen flow.

In a second step, an aqueous solution was prepared by dissolving **U­(DOTA)­(DMSO)** in a minimum amount of water and the **U­(DOTA)­(H**_**2**_ **O)** complex was subsequently precipitated by addition of tetrahydrofuran (THF). To ensure DMSO removal, this operation was repeated several times. The formation of **U­(DOTA)­(H**_**2**_ **O)** was confirmed by ^1^H NMR spectroscopy (see Figure S27). The final light green solid was redissolved in a minimal amount of water and repeatedly washed with THF. The biphasic mixture was centrifuged for 3 min at 3000 rpm, after which the upper THF phase was discarded. This washing–centrifugation cycle was repeated until the THF phase became completely colorless. Slow evaporation of this aqueous solution led to the deposition of crystals.

**Caution!** Natural uranium (mainly the isotope ^238^U) is a weak a-emitter (4.5 × 10^9^ years half-life). Synthesis of the **U­(DOTA)­(H_2_O)** complex has been carried out under fume hoods in laboratory LN1 of the ATALANTE facility. Recrystallization and physical measurements (CTM and single-crystal X-ray diffraction) were performed on the final compound (<1 kBq shipping) at the Chemistry Department of the University of Florence.

### Structural Characterization

Single-crystal X-ray diffraction (SCXRD) data of **U­(DOTA)­(H**_**2**_ **O)** were collected at 100 K using a Bruker D8 Venture diffractometer equipped with a PHOTON II detector and a microfocus source (Cu Kα radiation, λ = 1.54184 Å). Frames were collected with the Bruker APEX4 program suite, then integrated, and reduced with the Bruker SAINT software. The crystal structure was solved and refined using the Bruker SHELXTL software package. Crystallographic data and refinement parameters are reported in Table S1. SCXRD data have been deposited in the CCDC, and the deposition number is 2478981. Molecular plots were produced using the program Mercury^67^ and VESTA.^68^

### Cantilever Torque Magnetometry Measurements

Torque measurements were carried out on a Torque Magnetometry insert of a Quantum Design PPMS. A single-crystal sample of **U­(DOTA)­(H**_**2**_ **O)** was measured over a wide range of temperatures (2–250 K) and magnetic fields (0–9 T). During each measurement, the crystal was rotated by 180° around an axis perpendicular to the external magnetic field.

### *Ab Initio* Calculations

Calculations were performed on the XRD structure using the MOLCAS 7.8 package.^69^ The positions of the hydrogen atoms were optimized by using B3LYP KS-DFT.

The spin-free complete-active-space self-consistent field (SF-CASSCF) calculations^70^ were performed with the active space being the seven 5f orbitals and the associated electrons *i.e.*, CAS (2, 7). In the state-averaged calculations, we used 21 triplets and 27 singlets. Subsequently, the dynamical correlation was added using the multi state complete-active-space perturbation theory at the second-order (MS-CASPT2) method^71^ with a level shift of 0.3 au and 63 frozen orbitals. As shown previously,^72^ it is important to correlate actinide 5d orbitals. Calculations were done with the ANO-RCC basis sets,^73^ TZP for U, O, and N atoms, DZP for C, and DZ for H atoms. The scalar relativistic effects were accounted for by the means of the Douglas–Kroll–Hess transformation.^74–76^ In the second phase of the calculations, spin–orbit coupling was introduced via state interactions between the previously calculated spin-free states using the restricted active space state interaction method^77^ (RASSI) leading to the SO-MS-CASPT2 states. The spin–orbit (SO) integrals are calculated using the atomic mean-field integral (AMFI) approximation.^78^ The susceptibility tensor is calculated according to the literature.^79^

Calculations for the **Es­(DOTA)­(H**_**2**_ **O)** were done by a simple substitution of the U in the XRD **U­(DOTA)­(H_2_O)** structure with Es. In the state-averaged calculations, we used 21 sextets, 48 quartets, and 35 doublets. The dynamical correlation was added with the same level shift as in **U­(DOTA)­(H2O)** with the same number of frozen orbitals. The same basis sets were used as in the **U­(DOTA)­(H2O)**. SO-MS-CASPT2 was calculated from RASSI, and the tensor was calculated in the same manner as the U.

The crystal-field parameters were calculated based on the AILFT^80^ using the ORCA 5.0.3 quantum chemistry package,^81^ with SARC-DKH-DEF2-TZVP­(-f) basis sets^82,83^ and AUTOAUX features^84^ to automatically generate auxiliary basis sets for the resolution of identity approximation (RI-JK).^85^ Scalar relativistic effects were accounted for by using the second-order scalar relativistic Douglas Kroll Hess (DKH2) Hamiltonian formalism. The CASSCF calculation was performed with CAS­(2,7) with 21 triplets. Three structures were considered: the **U­(DOTA)** (without its apical water molecule), symmetrized to the C4 point group, the symmetric **U­(DOTA)­(H**_**2**_ **O)**, and the **U­(DOTA)­(H**_**2**_ **O)** in its XRD structure.

### Fitting Procedure

CTM data were fitted to extract the magnetic susceptibility tensor components as described in the text. The fit was performed with a homemade script in MATLAB (R2025a) using a machine-learning approach based on Gaussian process regression (*fitrgp*) combined with a lower confidence bound (LCB) acquisition function μ – 2σ. At each iteration, the model error was quantified as 1 – *R* ^2^, defined as^86^

$$\mathrm{err}=1-R^{2}=\frac{\sum_{i=1}^{n}(Y_{i}-S_{i}{)}^{2}}{\sum_{i=1}^{n}(\overset{®}{Y}-Y_{i}{)}^{2}}$$

where the summation runs over the number of points *n*, *Y* is the experimental data vector, *Y̅* its mean value, and *S* the simulated data vector. The parameter space was sampled with 5000 trial points generated by *Latin hypercube sampling* (*lhsdesign*), which provides a more uniform and efficient coverage of the multidimensional space than purely random sampling. The model selected the next point to evaluate according to the LCB function. Optimization proceeded until the error dropped below a set threshold of 0.01 or the maximum number of iterations (100) was reached. This machine-learning strategy was employed to efficiently explore the parameter space and avoid stagnation in local minima.

## Supplementary Material



## Notes

EPR spectra, crystal mounting and information regarding CTM, more CTM curves and fittings, NMR spectra, crystal information, crystal field parameters (PDF)
