# Supplementary material for: Determination of Magnetic Anisotropy Tensors in Actinide Complexes Using Torque Magnetometry: A U(IV) Case Study
Source: J Am Chem Soc. 2025 Dec 18;148(1):1106–15. doi: 10.1021/jacs.5c16992 (PMC12814344; doi:10.1021/jacs.5c16992)
Supplement: Supplementary file 1 [file ja5c16992_si_001.pdf]

Supplementary information

Determination of magnetic anisotropy tensors in actinide complexes using torque magnetometry: a U(IV) case study

Leonardo Tacconi[a£], Victor Adebayo[b£], Laura Chelazzi[c], Claude Berthon[d], Hélène Bolvin*[b], Mauro Perfetti*[a]

a. Department of Chemistry "Ugo Schiff", University of Florence and INSTM Research Unit, Via della Lastruccia 3-13, 50019 Sesto Fiorentino (FI), Italy.
b. Laboratoire de Chimie et Physique Quantiques, CNRS, Université de Toulouse, 31062 Toulouse, France.
c. Centro di Servizi di Cristallografia Strutturale, CRIST, 50019 Sesto Fiorentino (FI), Italy.
d. CEA, DES, ISEC, DMRC, Université Montpellier, Bagnols-sur-Cèze 30207, France.

* mauro.perfetti@unifi.it; bolvin@irsamc.ups-tlse.fr

[£] These authors equally contributed to this work.

Supplementary Figures

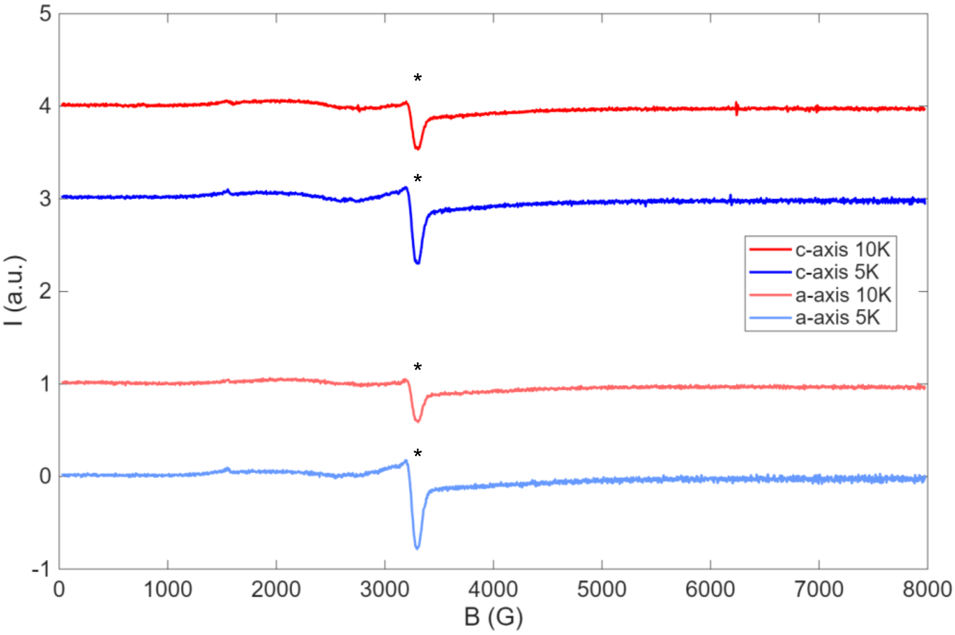

Figure S1 – Experimental X-band EPR signals acquired at 10 and 5 K along two different crystallographic orientations on a single crystal of **U(DOTA)(H_2O)**. The peak observed around *g* = 2 and marked with an asterisk, is attributed to the cavity used during the experiments.

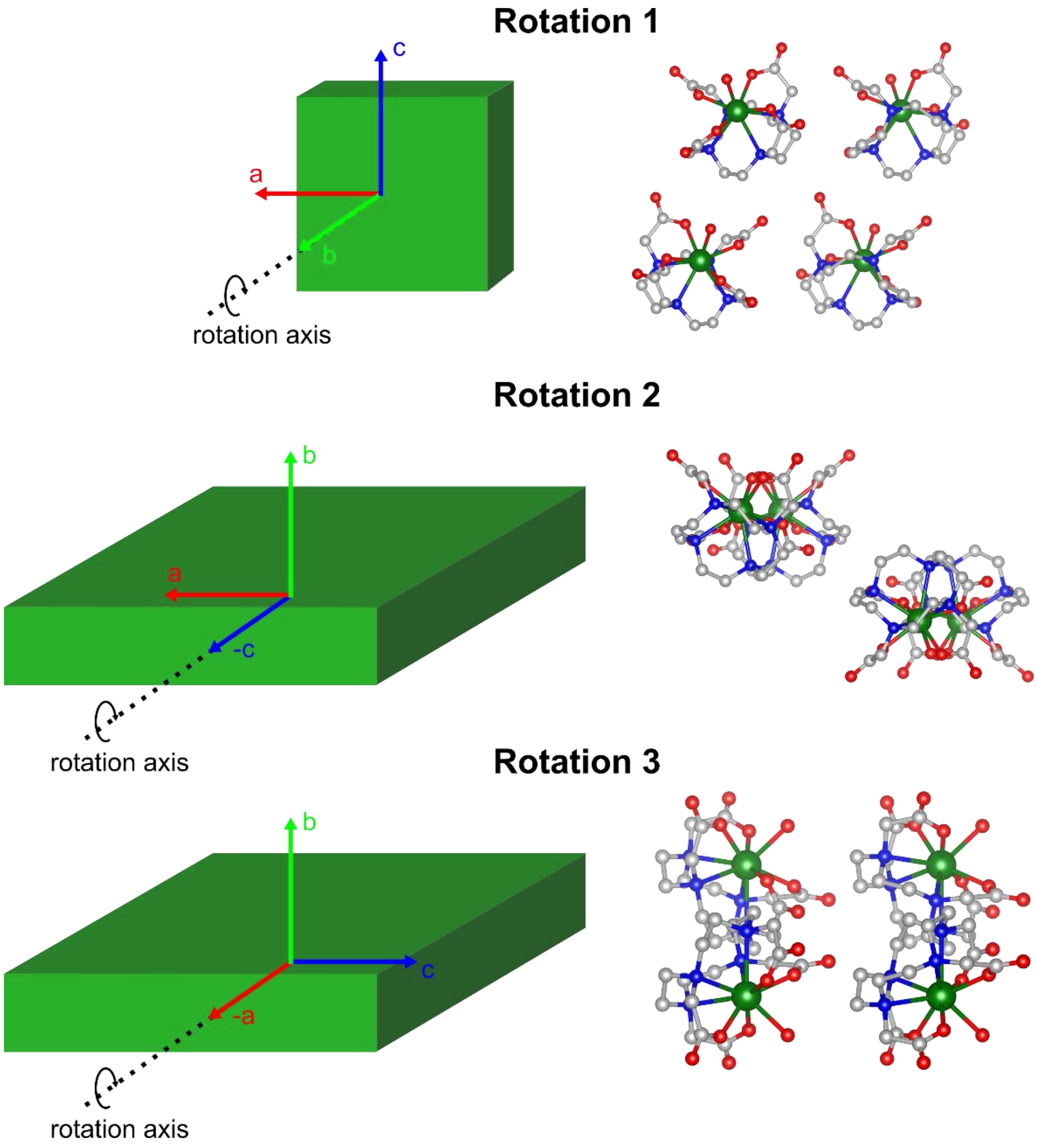

Figure S2 – Experimental rotations performed during cantilever torque magnetometry measurements, sample when the rotation axis θ is equal to 0°. For each rotation the magnetic field is applied vertically. On the right column are depicted the orientations of the **U(DOTA)(H_2O)** molecules at the beginning of each rotation.

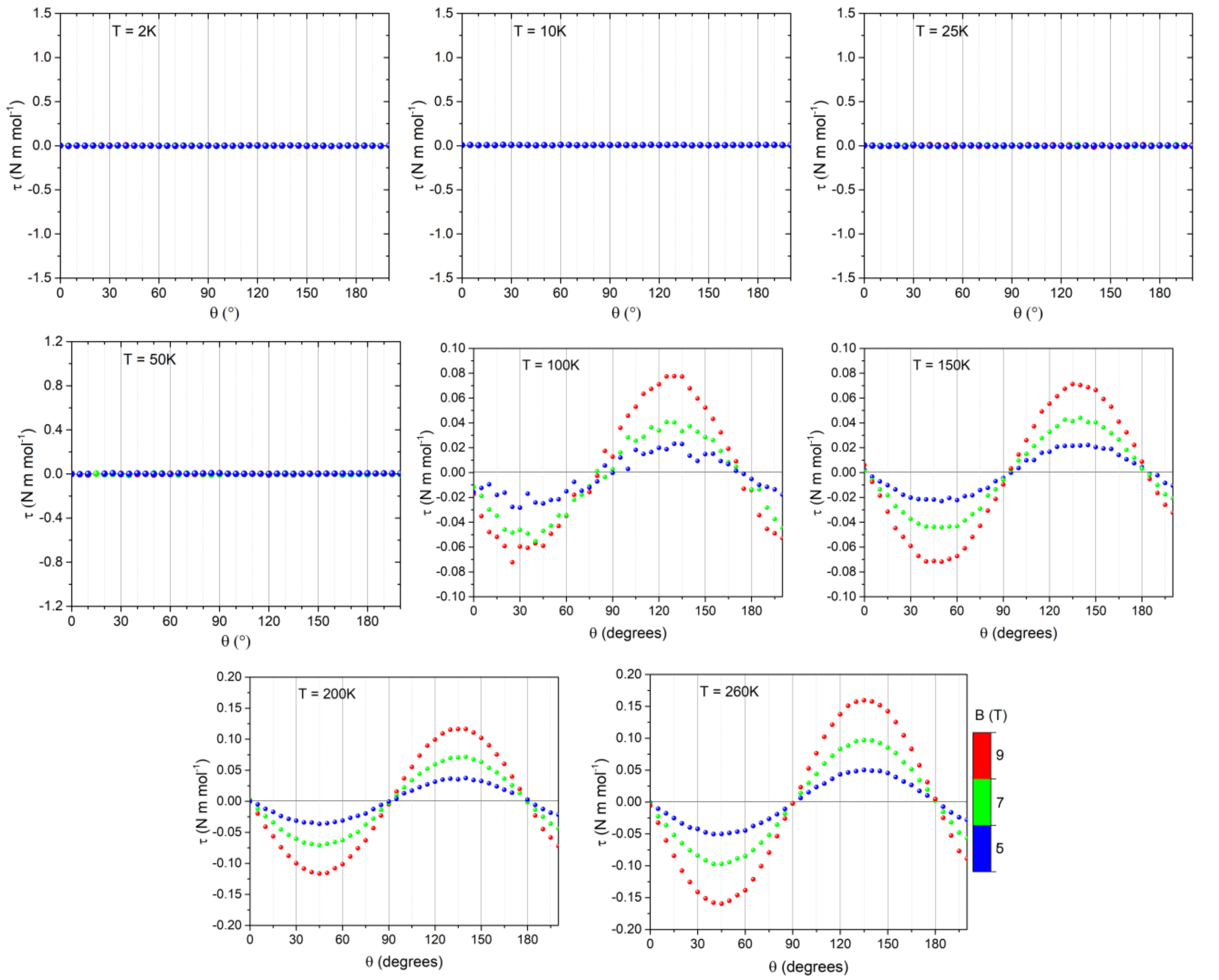

Figure S3 – Cantilever torque magnetometry results obtained during Rotation 1 at various temperatures and magnetic fields.

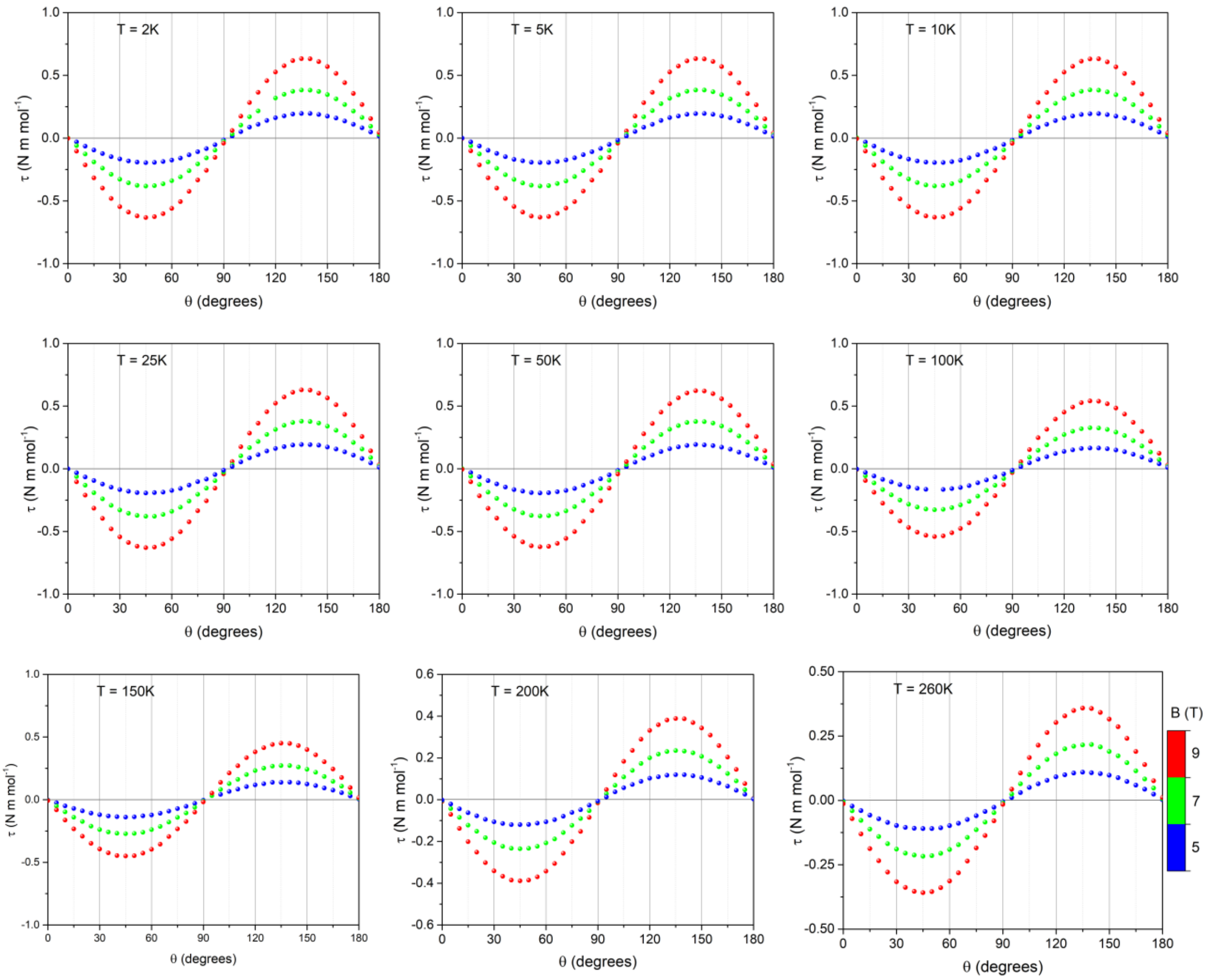

Figure S4 – Cantilever torque magnetometry results obtained during Rotation 2 at various temperatures and magnetic fields.

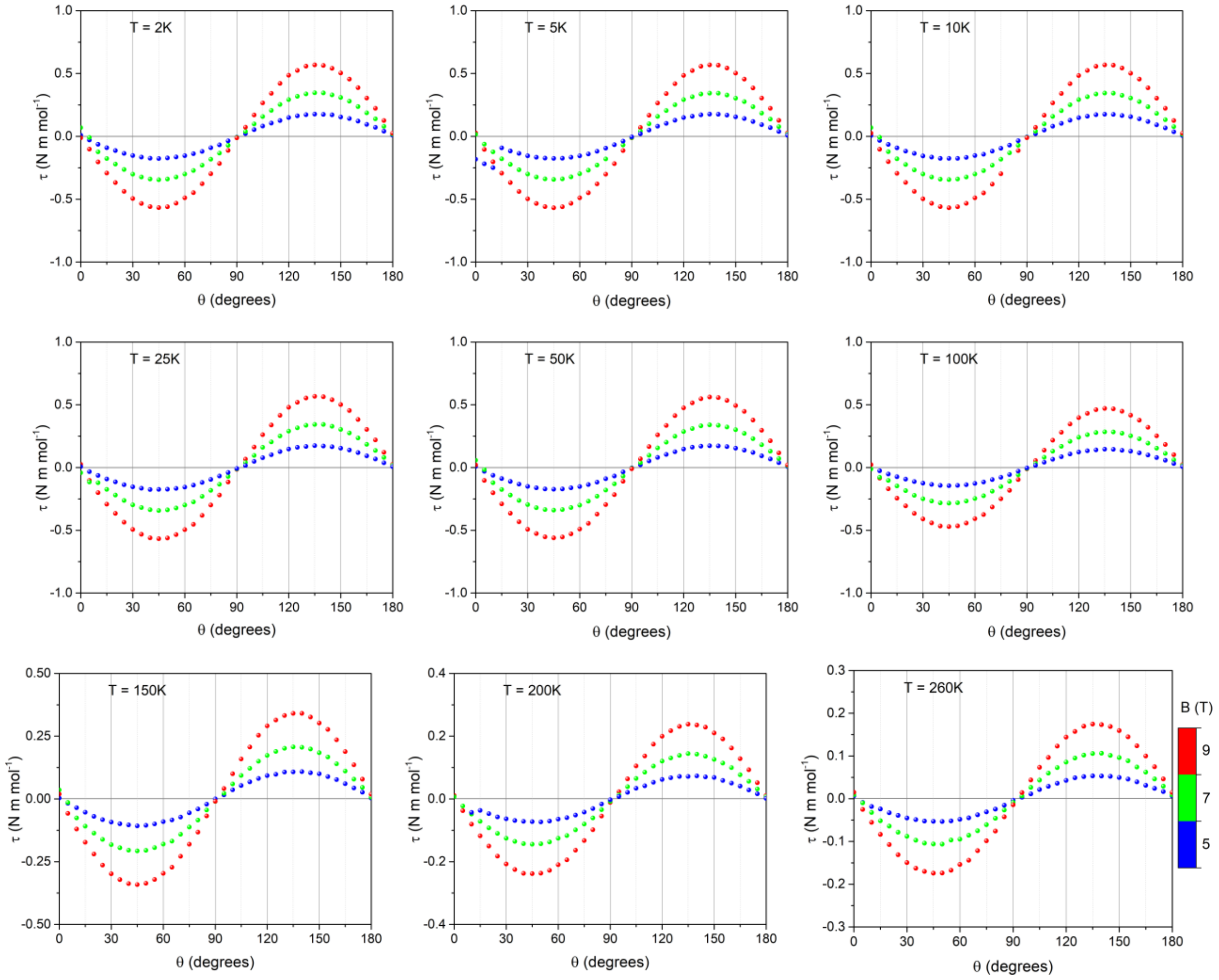

Figure S5 – Cantilever torque magnetometry results obtained during Rotation 3 at various temperatures and magnetic fields.

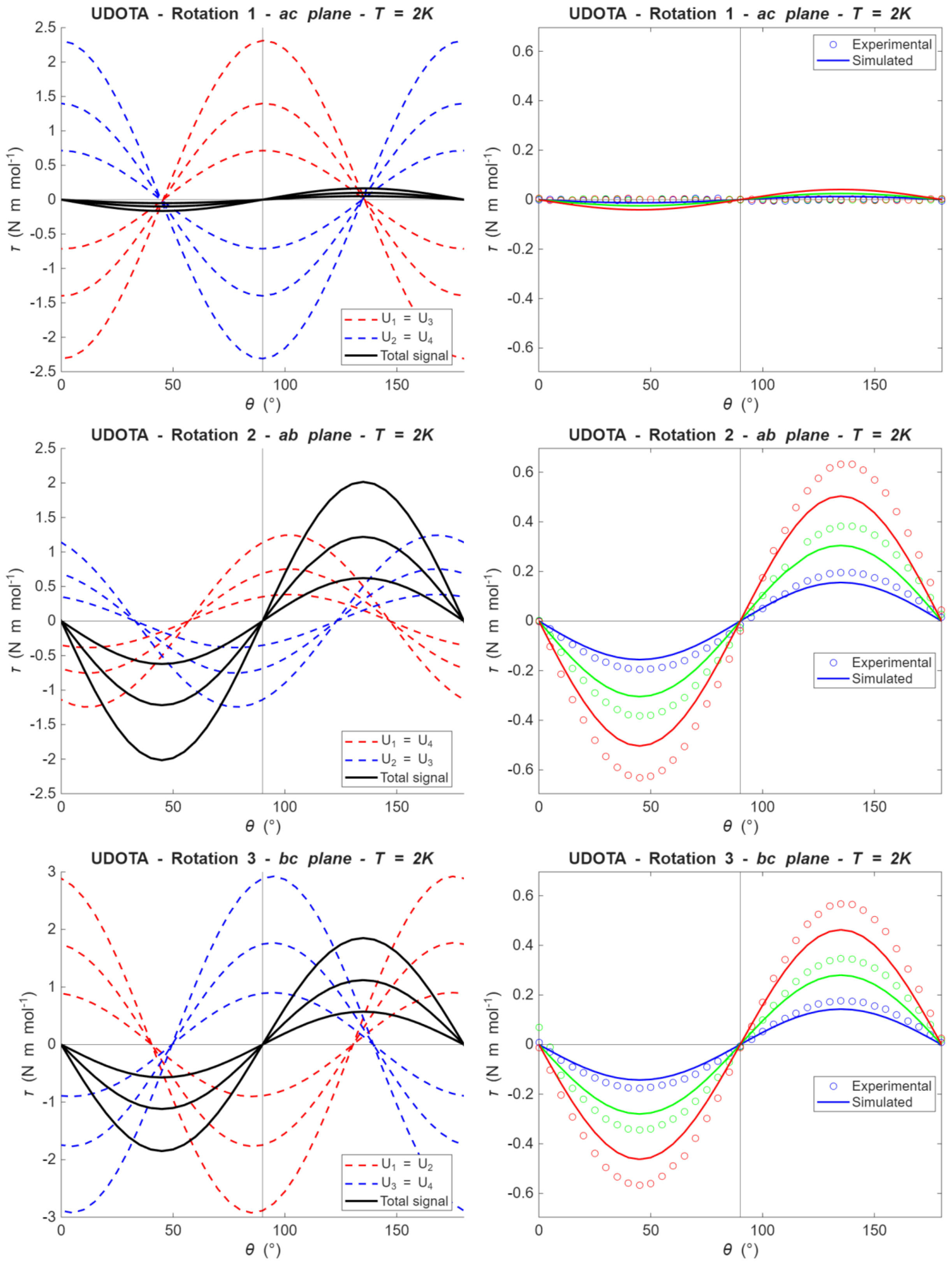

Figure S6 – Left column: contributions of each of the four uranium centres to the total torque signal. Right column: experimental and simulated cantilever torque magnetometry curves obtained at 2K and different magnetic fields: 9T (red), 7T (green) and 5T (blue). Simulations were carried out with parameters obtain from *ab initio* calculations, as discussed in main.

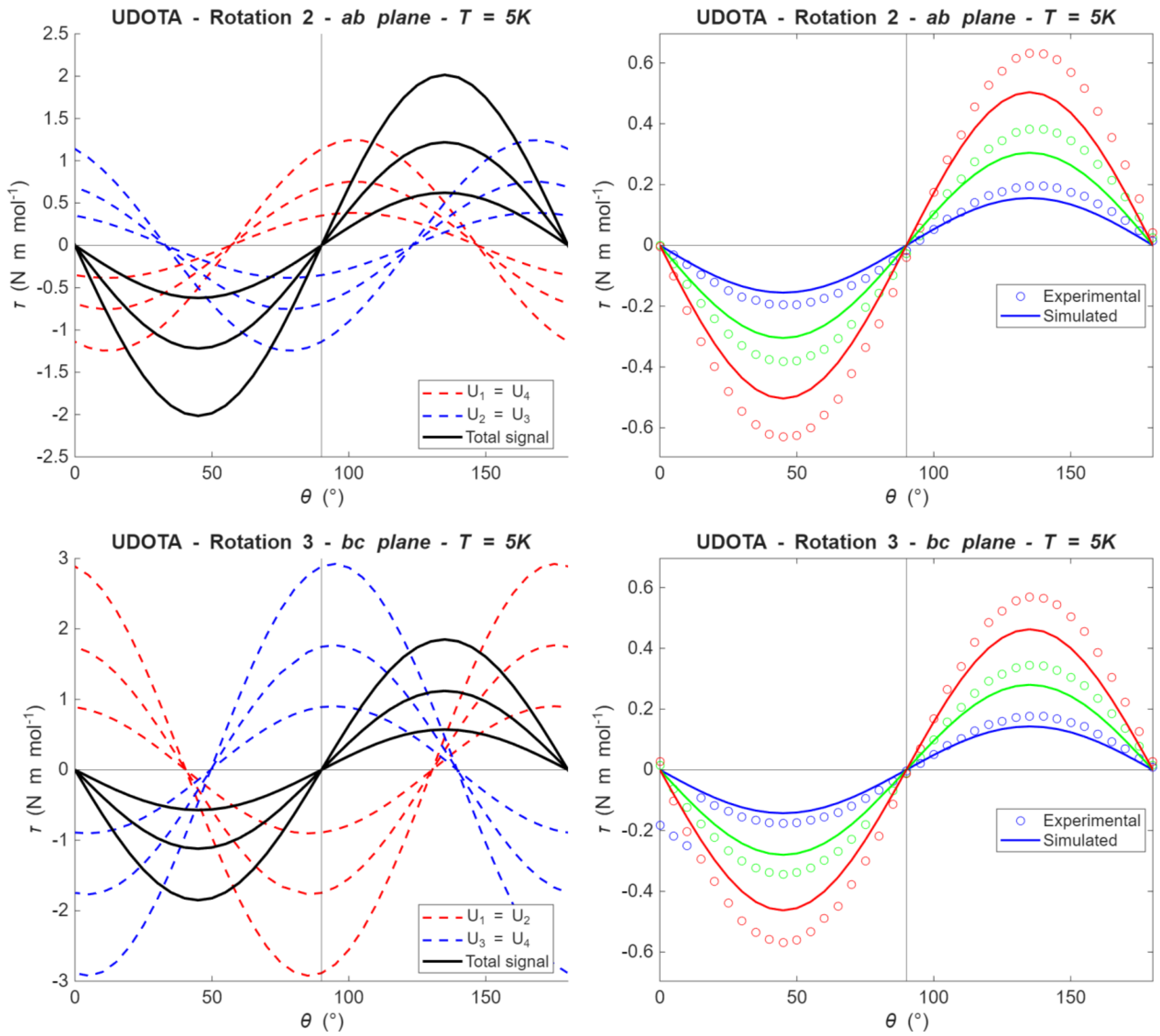

Figure S7 – Left column: contributions of each of the four uranium centres to the total torque signal. Right column: experimental and simulated cantilever torque magnetometry curves obtained at 5K and different magnetic fields: 9T (red), 7T (green) and 5T (blue). Simulations were carried out with parameters obtain from *ab initio* calculations, as discussed in main.

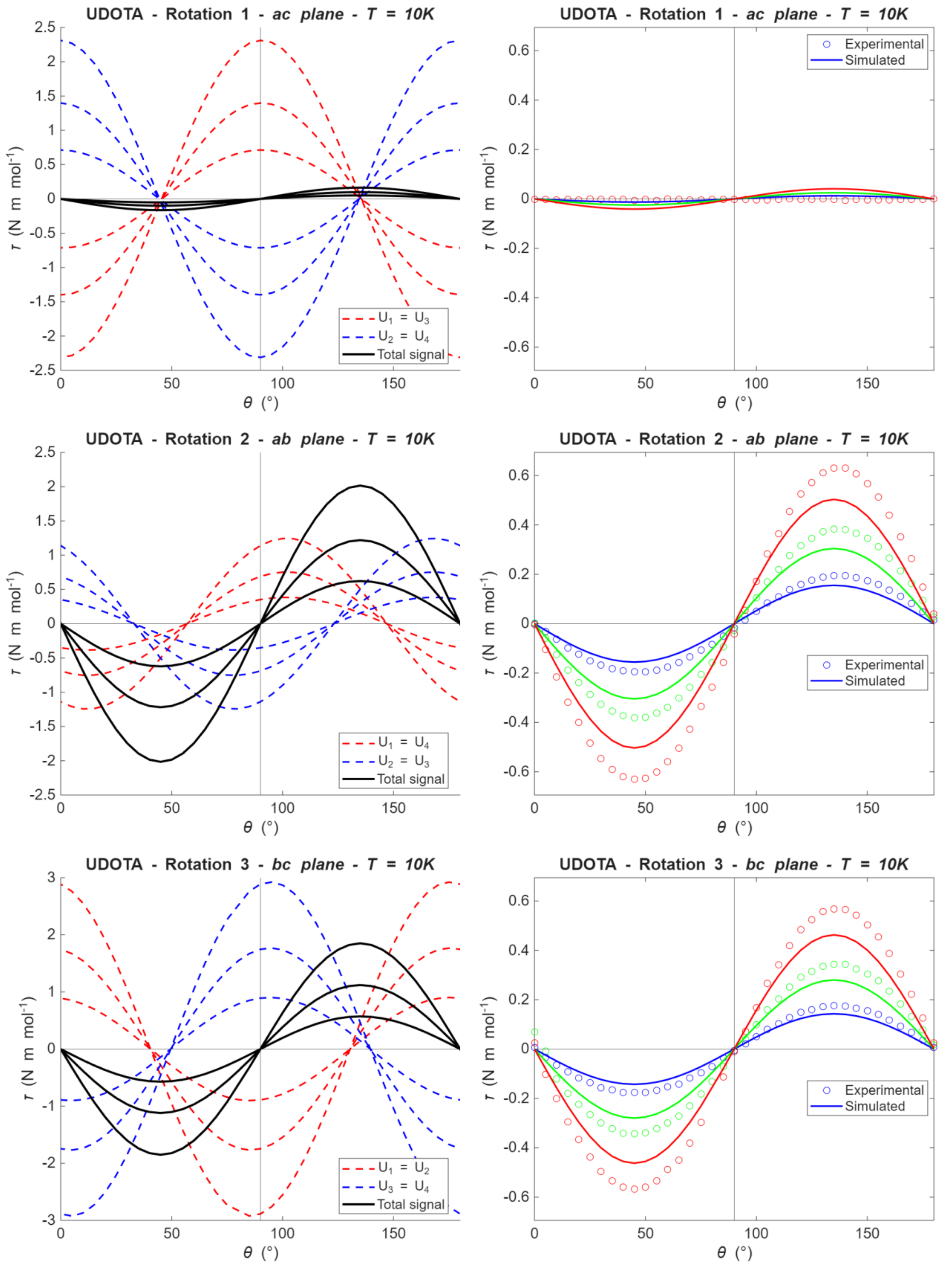

Figure S8 – Left column: contributions of each of the four uranium centres to the total torque signal. Right column: experimental and simulated cantilever torque magnetometry curves obtained at 10K and different magnetic fields: 9T (red), 7T (green) and 5T (blue). Simulations were carried out with parameters obtain from *ab initio* calculations, as discussed in main.

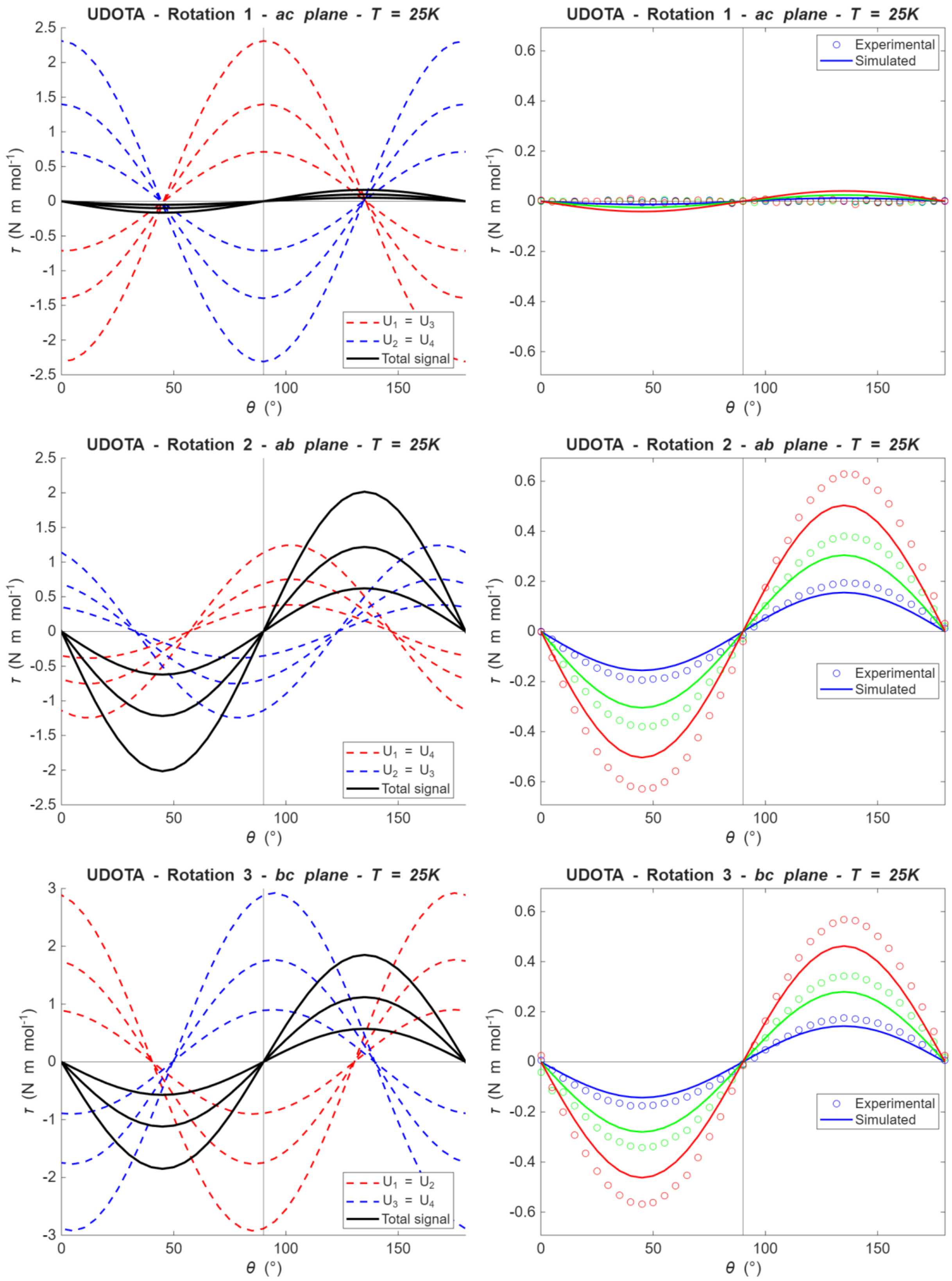

Figure S9 – Left column: contributions of each of the four uranium centres to the total torque signal. Right column: experimental and simulated cantilever torque magnetometry curves obtained at 25K and different magnetic fields: 9T (red), 7T (green) and 5T (blue). Simulations were carried out with parameters obtain from *ab initio* calculations, as discussed in main.

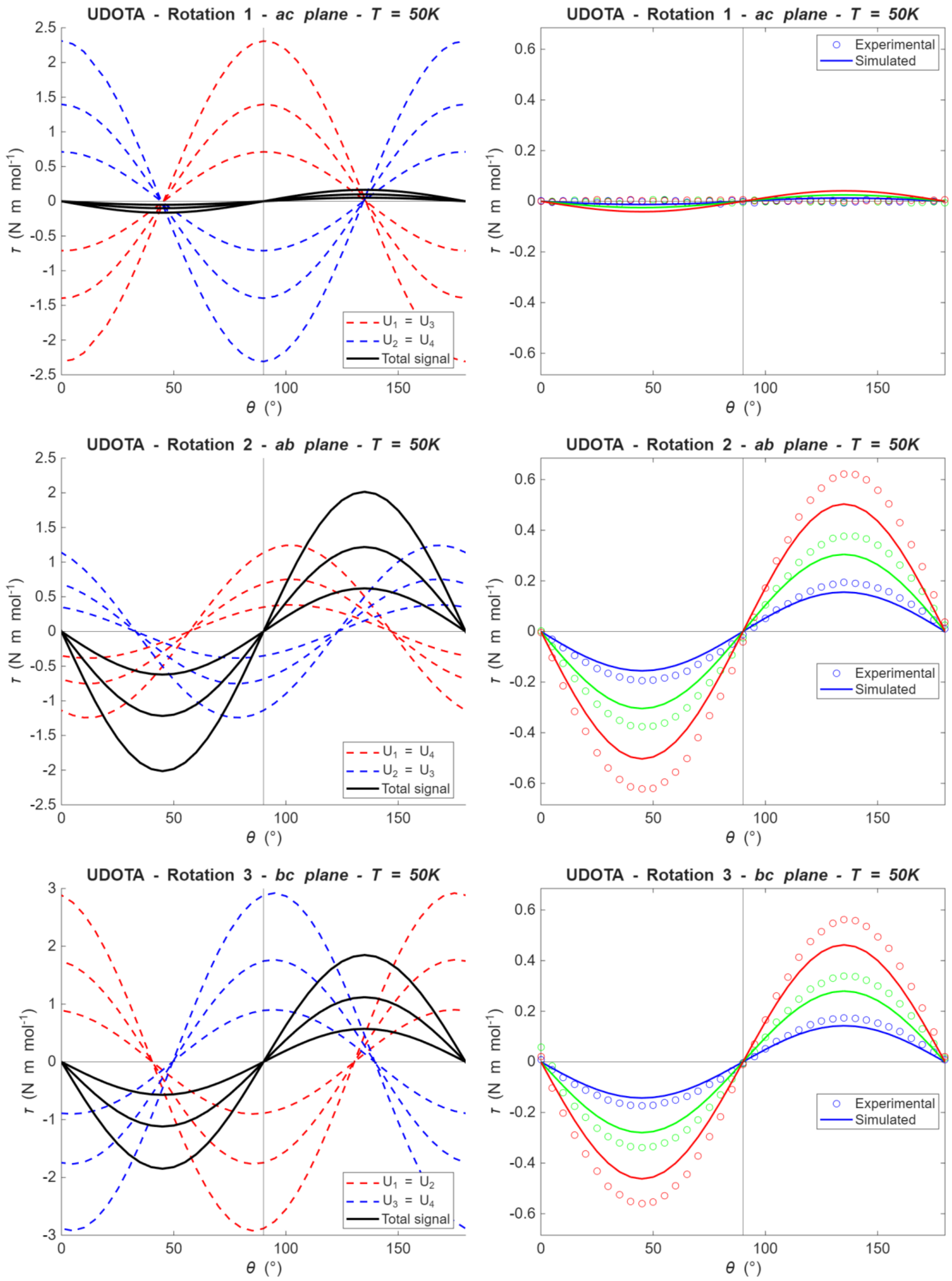

Figure S10 – Left column: contributions of each of the four uranium centres to the total torque signal. Right column: experimental and simulated cantilever torque magnetometry curves obtained at 50K and different magnetic fields: 9T (red), 7T (green) and 5T (blue). Simulations were carried out with parameters obtain from *ab initio* calculations, as discussed in main.

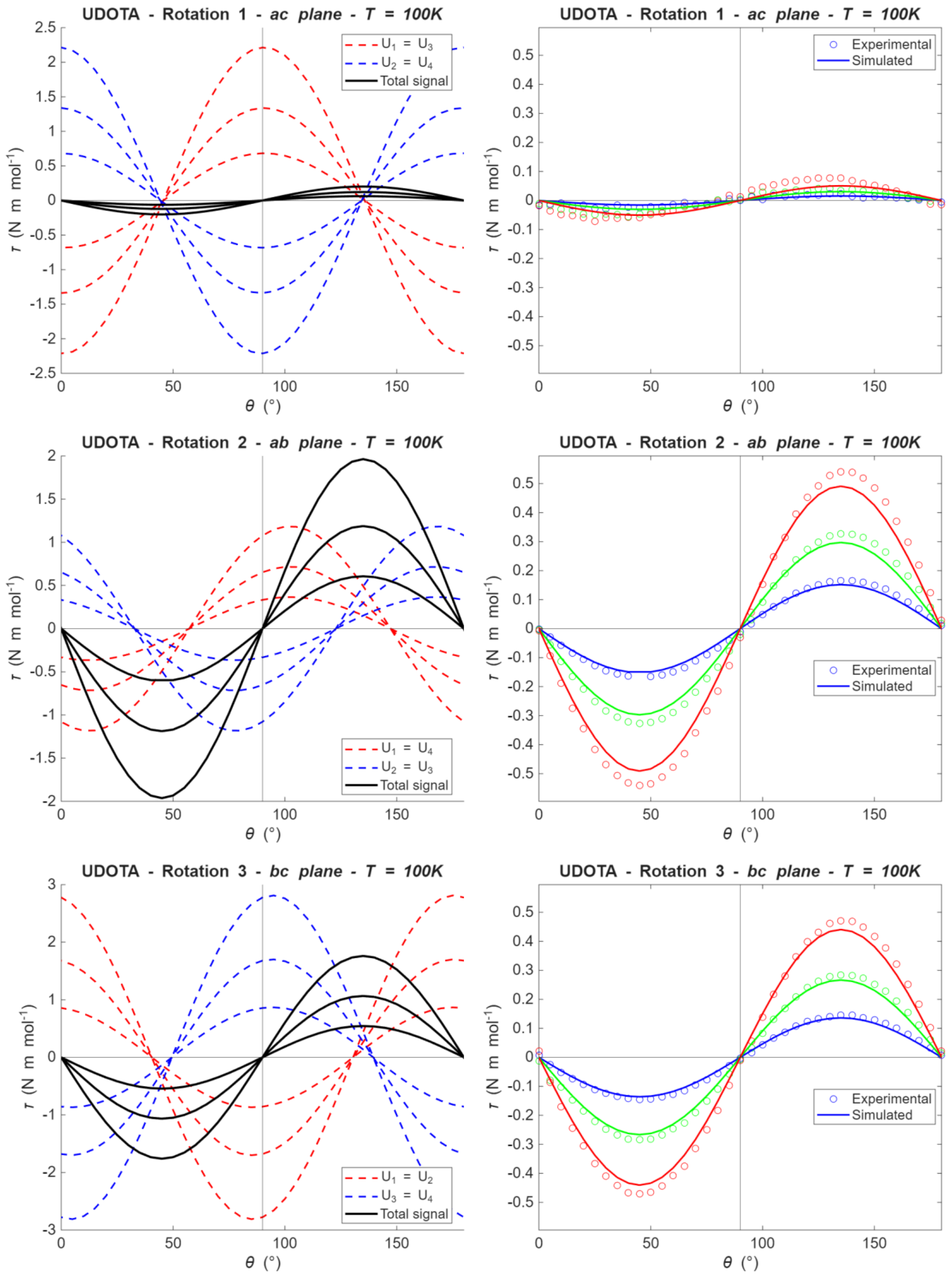

Figure S11 – Left column: contributions of each of the four uranium centres to the total torque signal. Right column: experimental and simulated cantilever torque magnetometry curves obtained at 100K and different magnetic fields: 9T (red), 7T (green) and 5T (blue). Simulations were carried out with parameters obtain from *ab initio* calculations, as discussed in main.

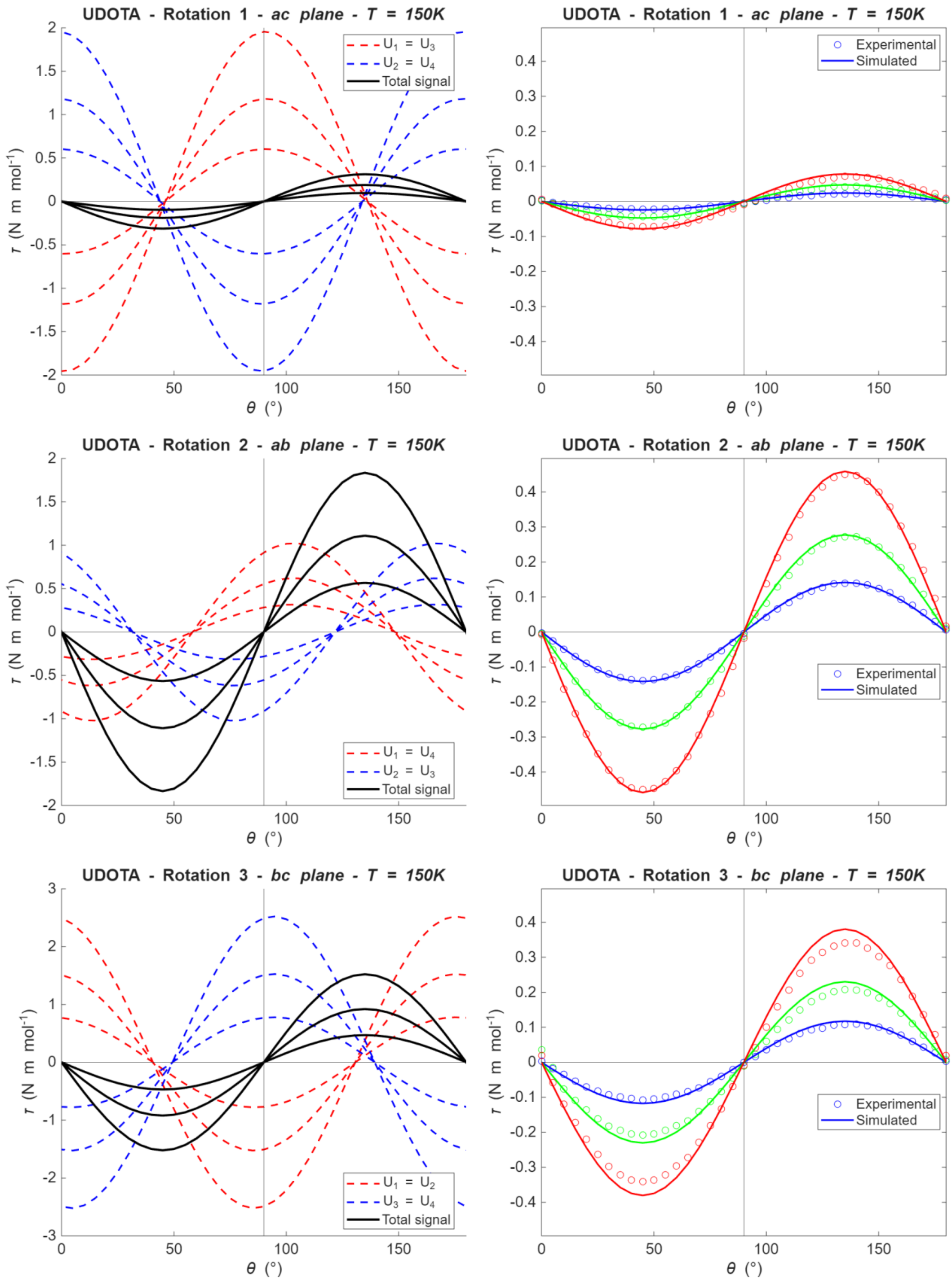

Figure S12 – Left column: contributions of each of the four uranium centres to the total torque signal. Right column: experimental and simulated cantilever torque magnetometry curves obtained at 150K and different magnetic fields: 9T (red), 7T (green) and 5T (blue). Simulations were carried out with parameters obtain from *ab initio* calculations, as discussed in main.

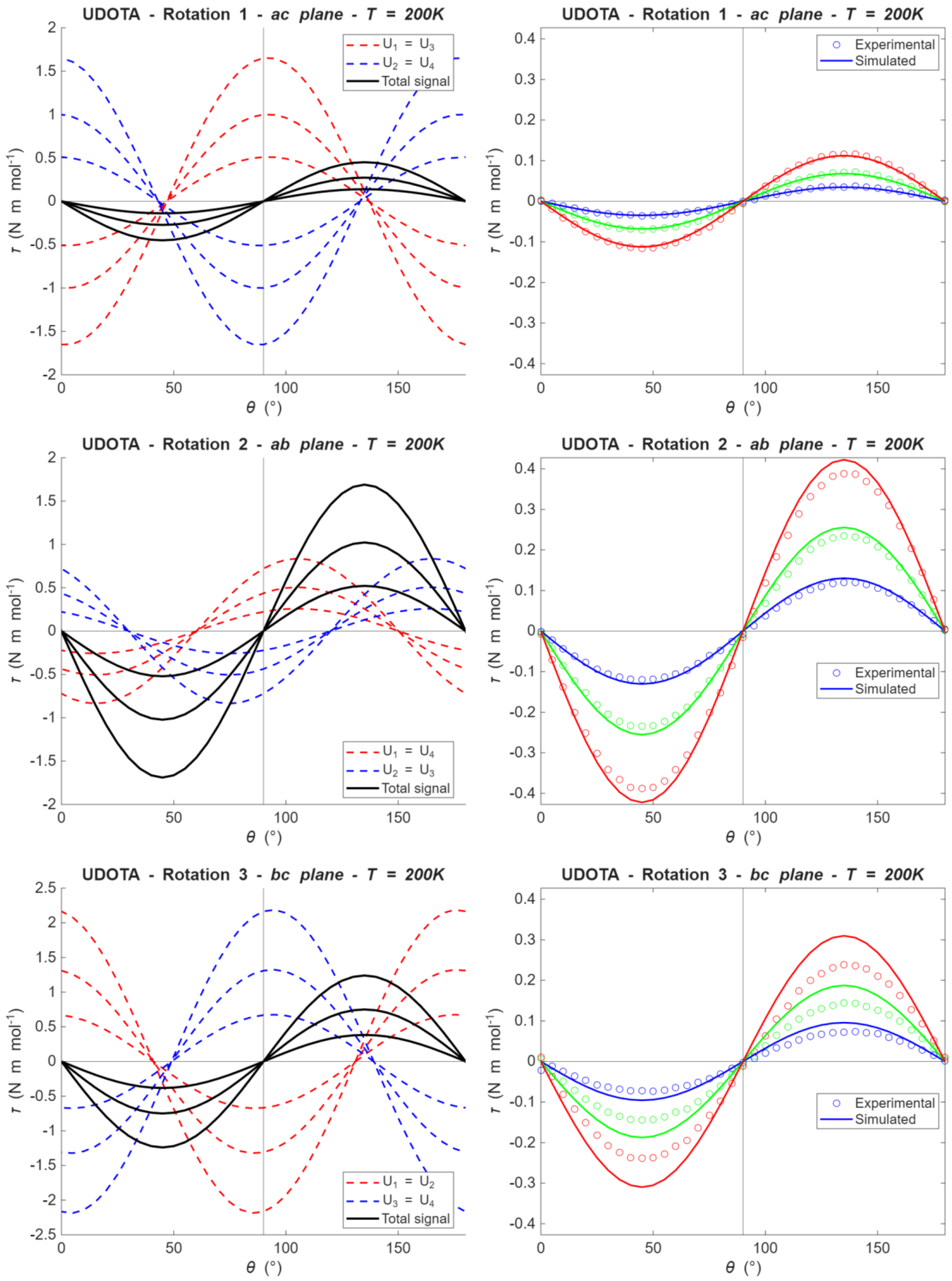

Figure S13 – Left column: contributions of each of the four uranium centres to the total torque signal. Right column: experimental and simulated cantilever torque magnetometry curves obtained at 200K and different magnetic fields: 9T (red), 7T (green) and 5T (blue). Simulations were carried out with parameters obtain from *ab initio* calculations, as discussed in main.

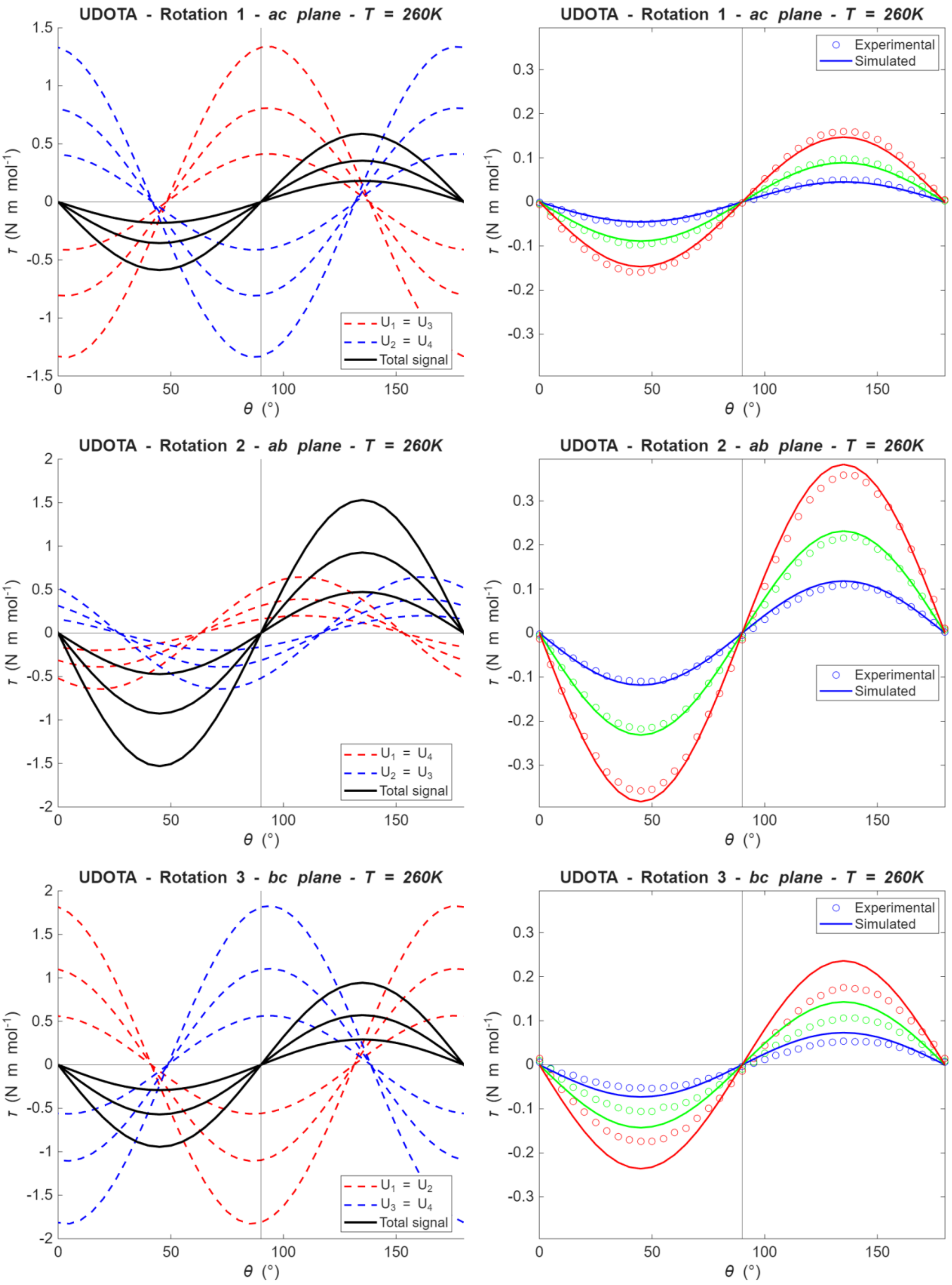

Figure S14 – Left column: contributions of each of the four uranium centres to the total torque signal. Right column: experimental and simulated cantilever torque magnetometry curves obtained at 260K and different magnetic fields: 9T (red), 7T (green) and 5T (blue). Simulations were carried out with parameters obtain from *ab initio* calculations, as discussed in main.

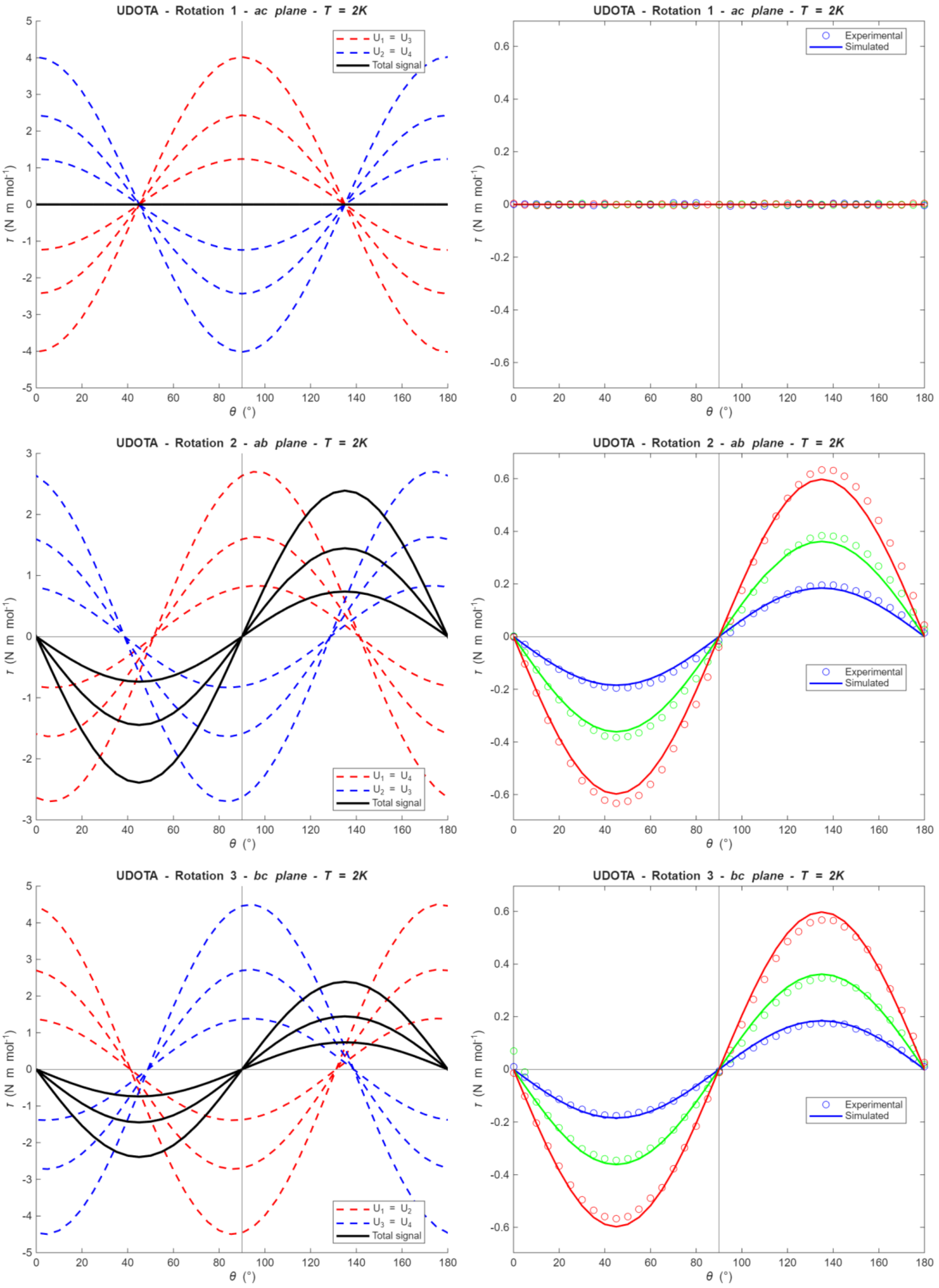

Figure S15 – Left column: contributions of each of the four uranium centres to the total torque signal. Right column: experimental and simulated cantilever torque magnetometry curves obtained at 2K and different magnetic fields: 9T (red), 7T (green) and 5T (blue). Simulations were carried out with parameters obtain from fitting procedure, as discussed in main.

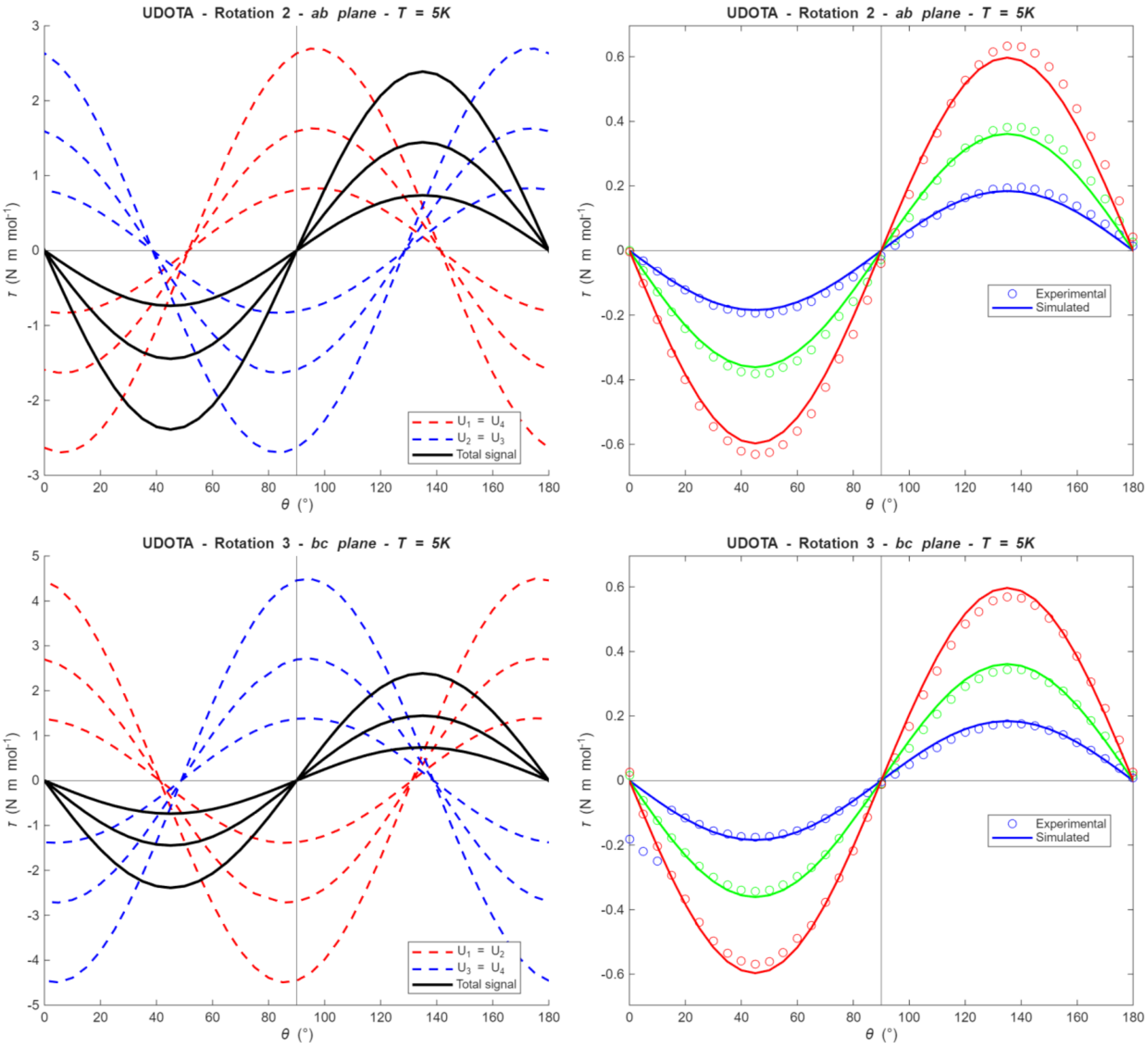

Figure S16 – Left column: contributions of each of the four uranium centres to the total torque signal. Right column: experimental and simulated cantilever torque magnetometry curves obtained at 5K and different magnetic fields: 9T (red), 7T (green) and 5T (blue). Simulations were carried out with parameters obtain from fitting procedure, as discussed in main.

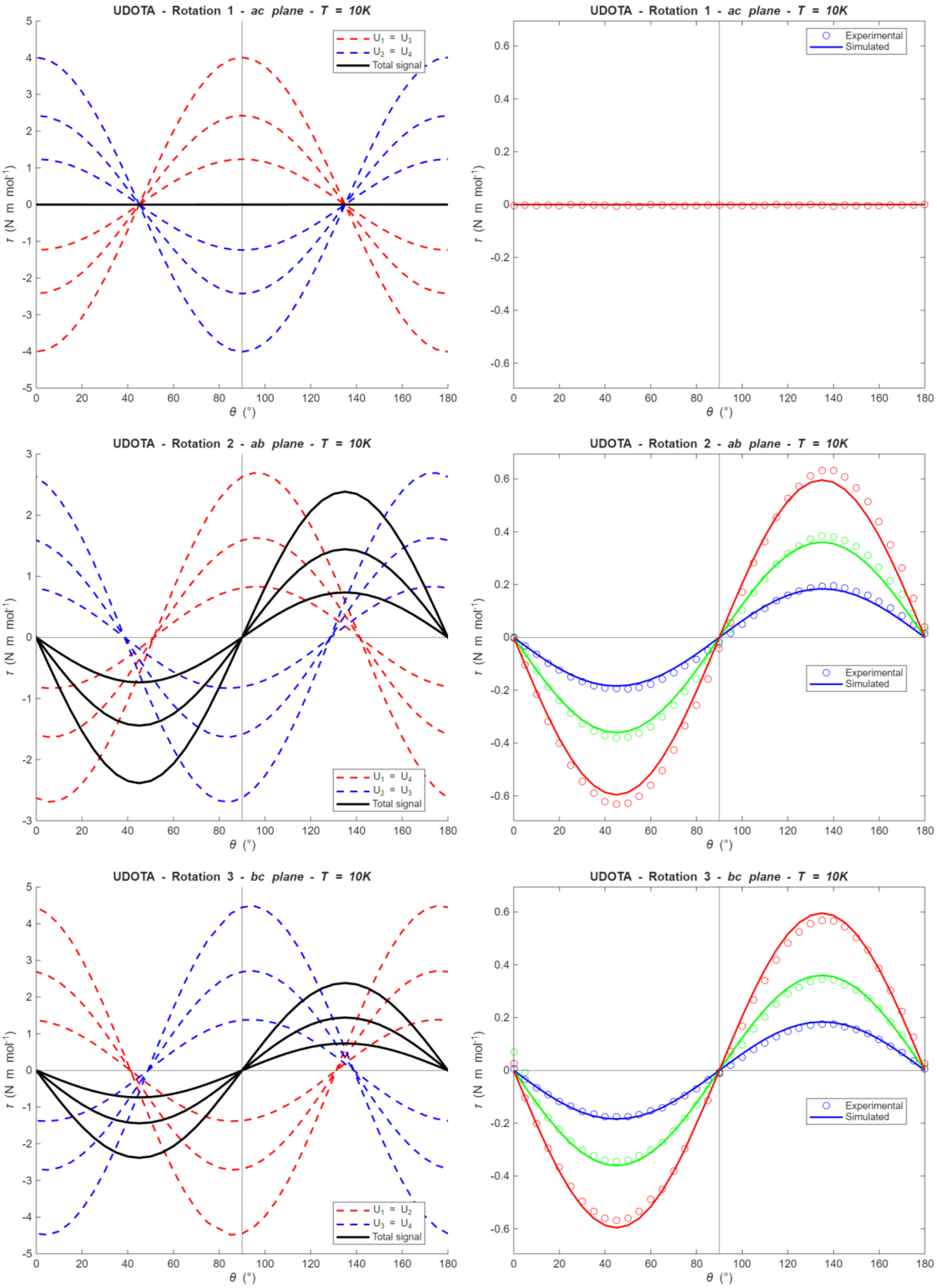

Figure S17 – Left column: contributions of each of the four uranium centres to the total torque signal. Right column: experimental and simulated cantilever torque magnetometry curves obtained at 10K and different magnetic fields: 9T (red), 7T (green) and 5T (blue). Simulations were carried out with parameters obtain from fitting procedure, as discussed in main.

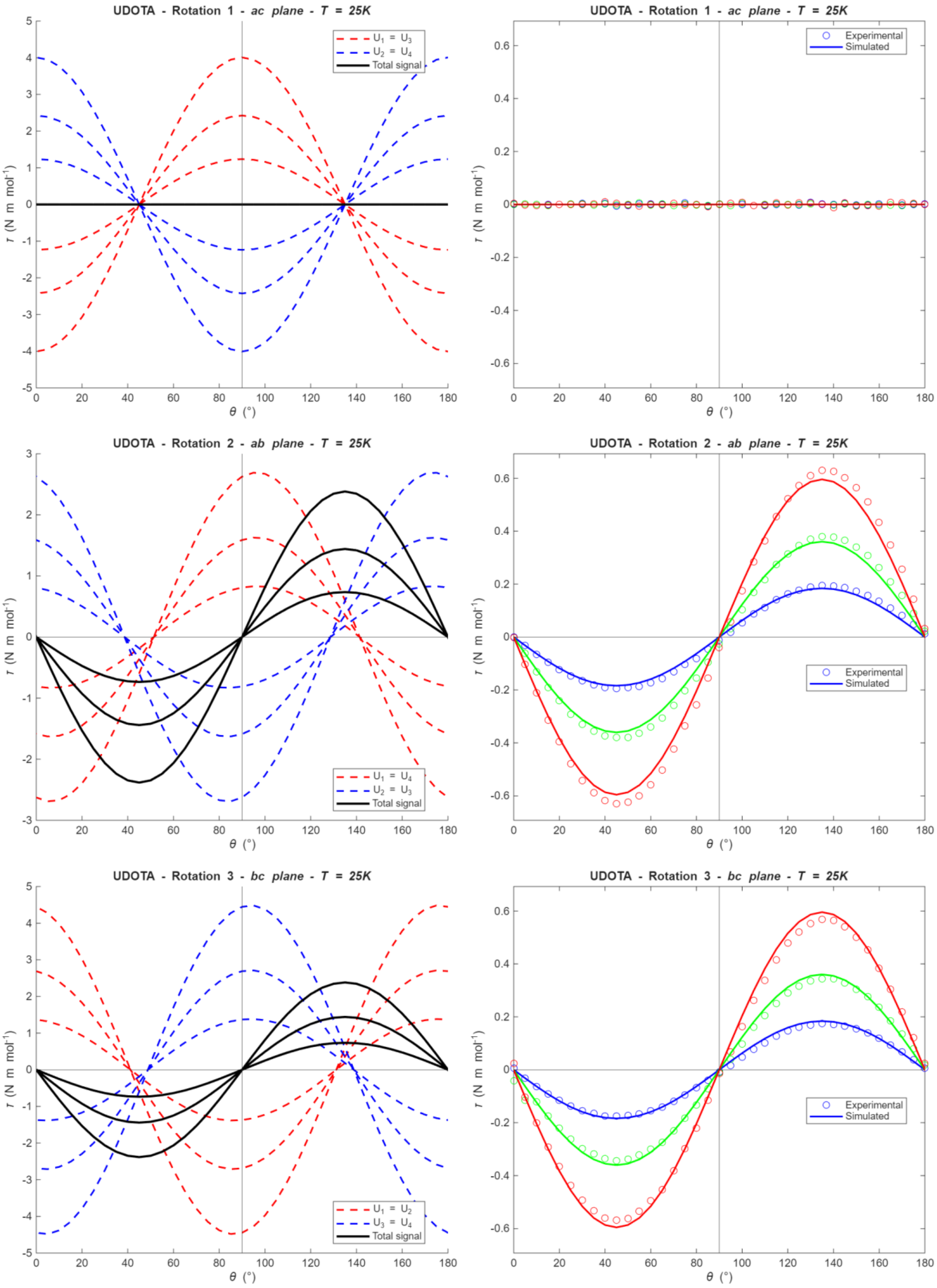

Figure S18 – Left column: contributions of each of the four uranium centres to the total torque signal. Right column: experimental and simulated cantilever torque magnetometry curves obtained at 25K and different magnetic fields: 9T (red), 7T (green) and 5T (blue). Simulations were carried out with parameters obtain from fitting procedure, as discussed in main.

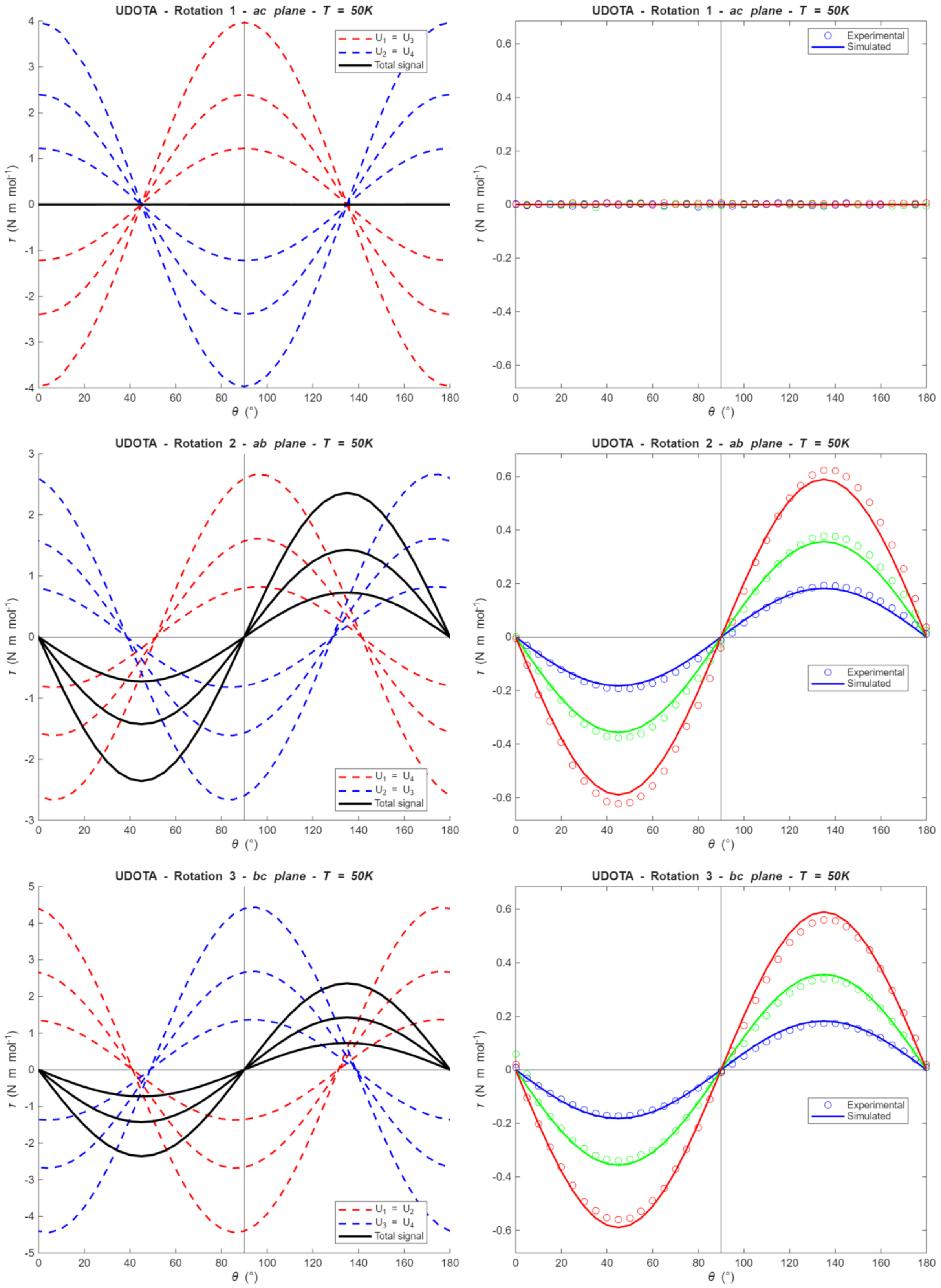

Figure S19 – Left column: contributions of each of the four uranium centres to the total torque signal. Right column: experimental and simulated cantilever torque magnetometry curves obtained at 50K and different magnetic fields: 9T (red), 7T (green) and 5T (blue). Simulations were carried out with parameters obtain from fitting procedure, as discussed in main.

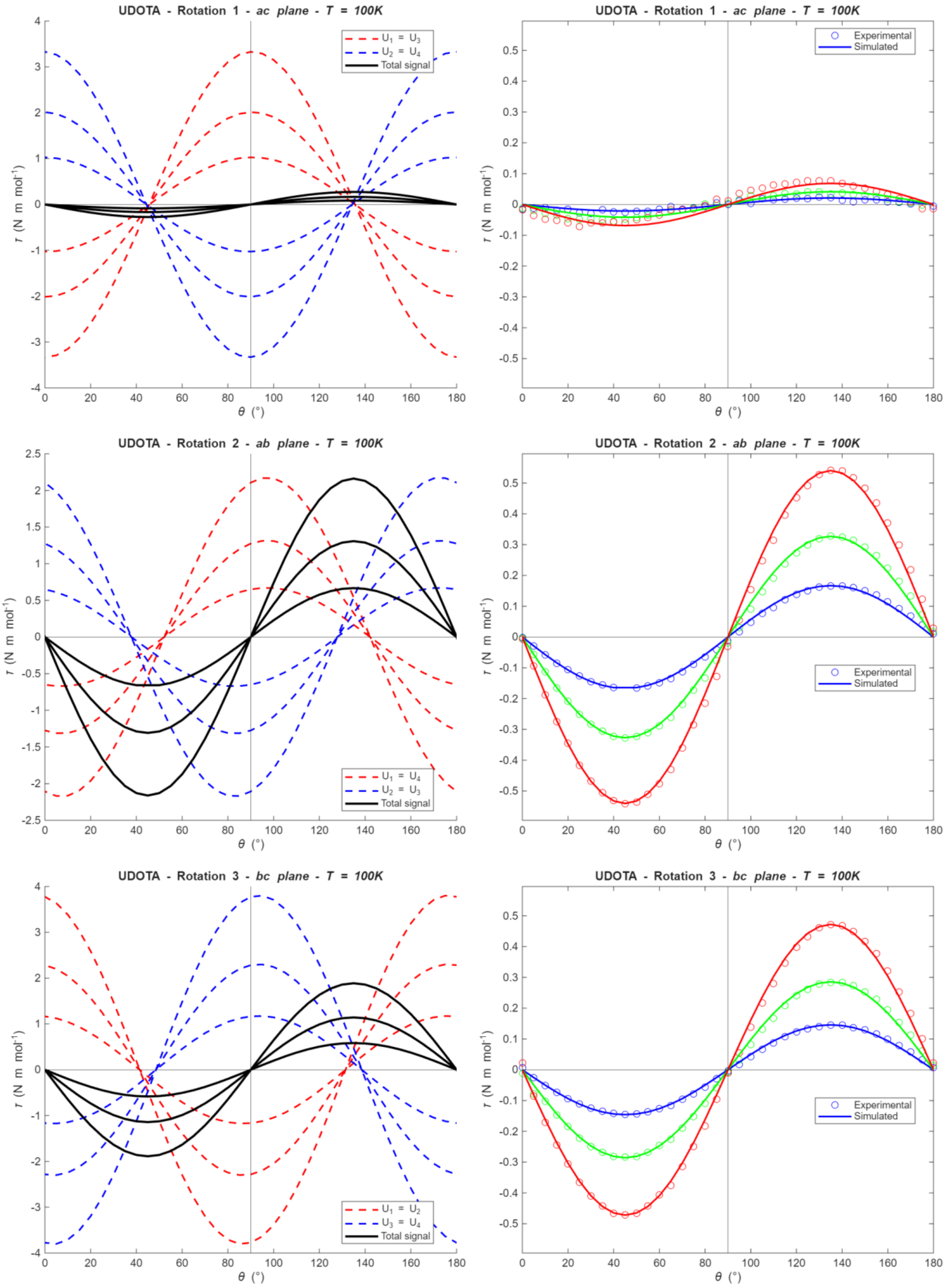

Figure S20 – Left column: contributions of each of the four uranium centres to the total torque signal. Right column: experimental and simulated cantilever torque magnetometry curves obtained at 100K and different magnetic fields: 9T (red), 7T (green) and 5T (blue). Simulations were carried out with parameters obtain from fitting procedure, as discussed in main.

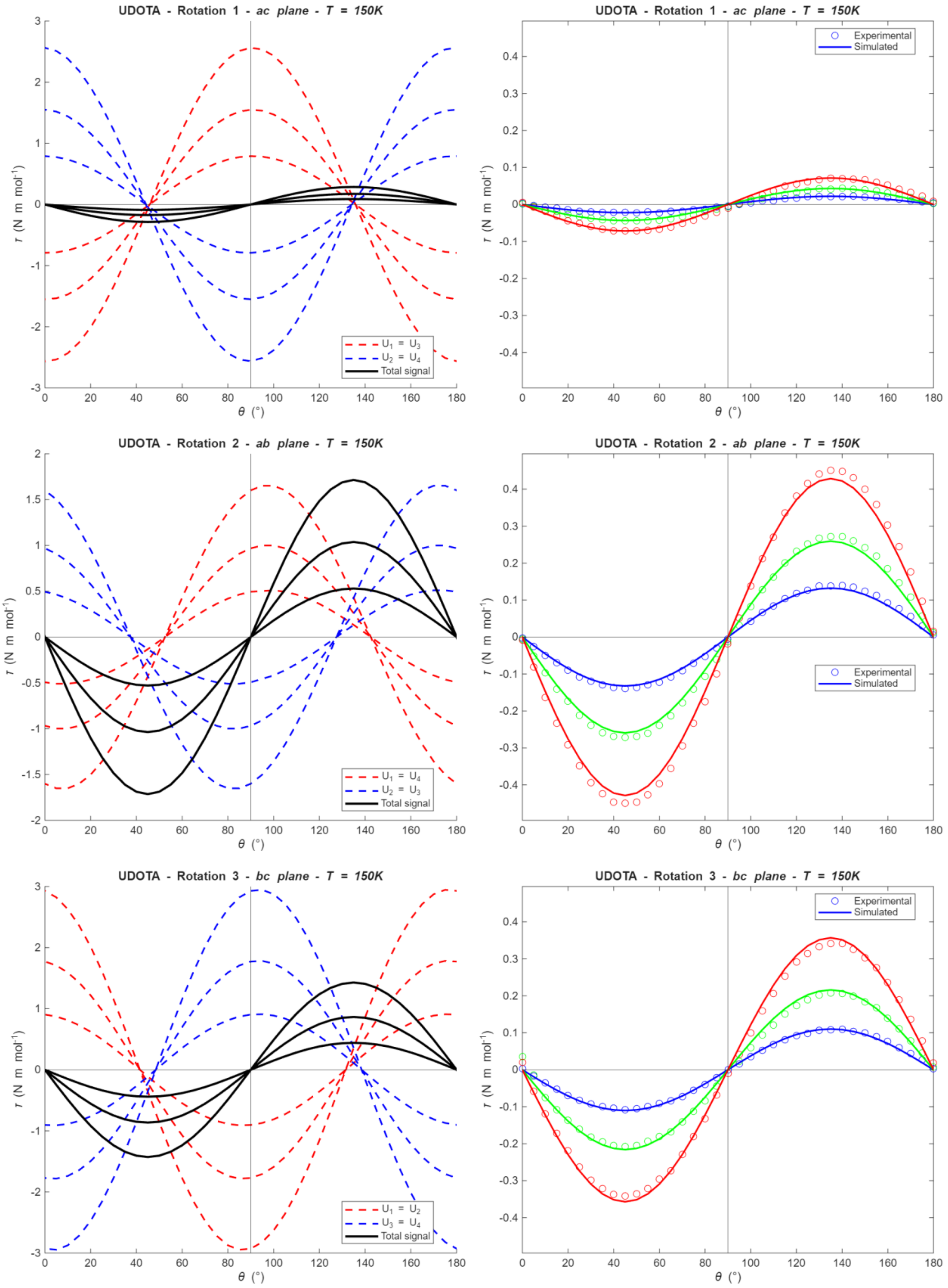

Figure S21 – Left column: contributions of each of the four uranium centres to the total torque signal. Right column: experimental and simulated cantilever torque magnetometry curves obtained at 150K and different magnetic fields: 9T (red), 7T (green) and 5T (blue). Simulations were carried out with parameters obtain from fitting procedure, as discussed in main.

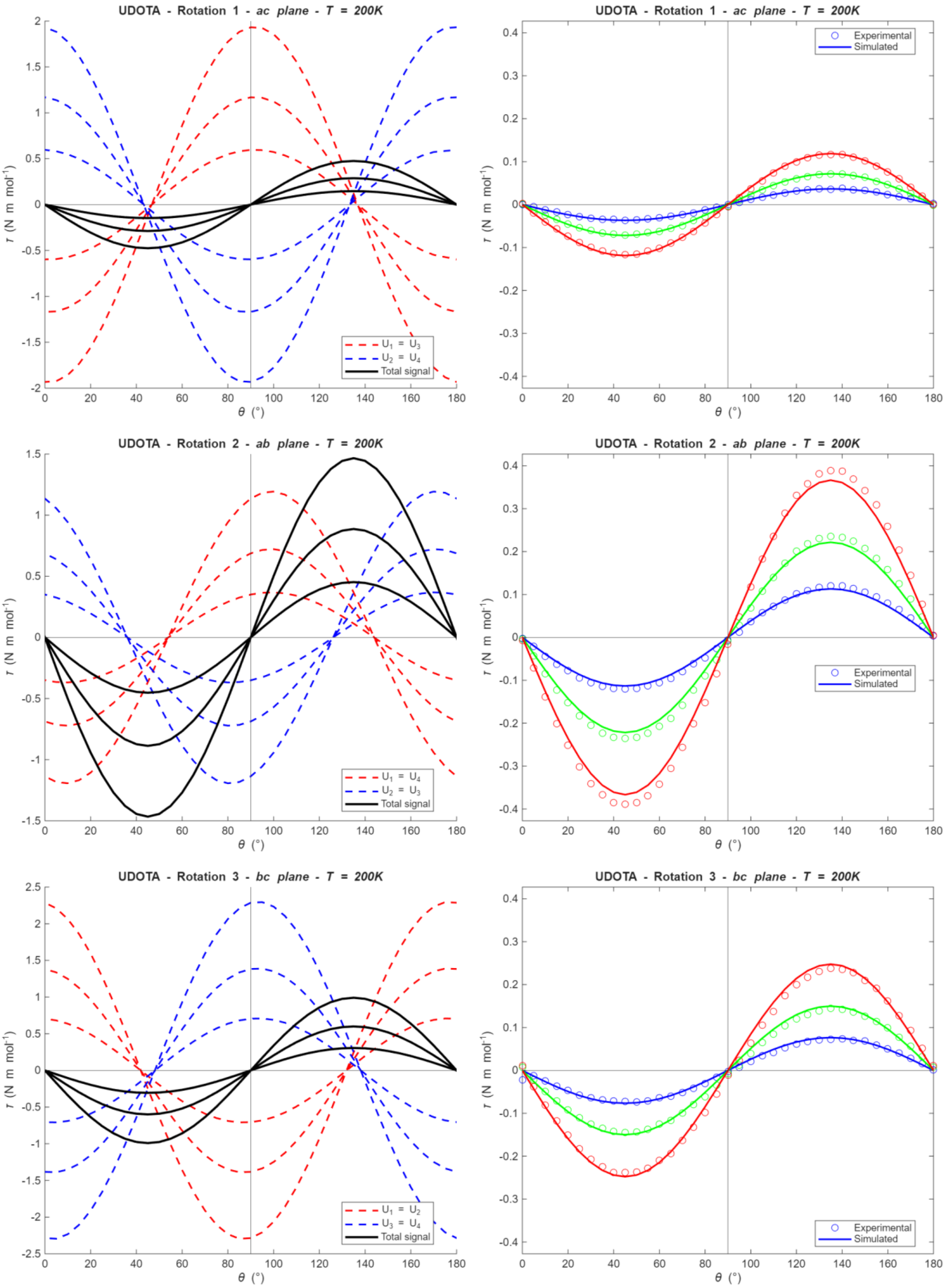

Figure S22 – Left column: contributions of each of the four uranium centres to the total torque signal. Right column: experimental and simulated cantilever torque magnetometry curves obtained at 200K and different magnetic fields: 9T (red), 7T (green) and 5T (blue). Simulations were carried out with parameters obtain from fitting procedure, as discussed in main.

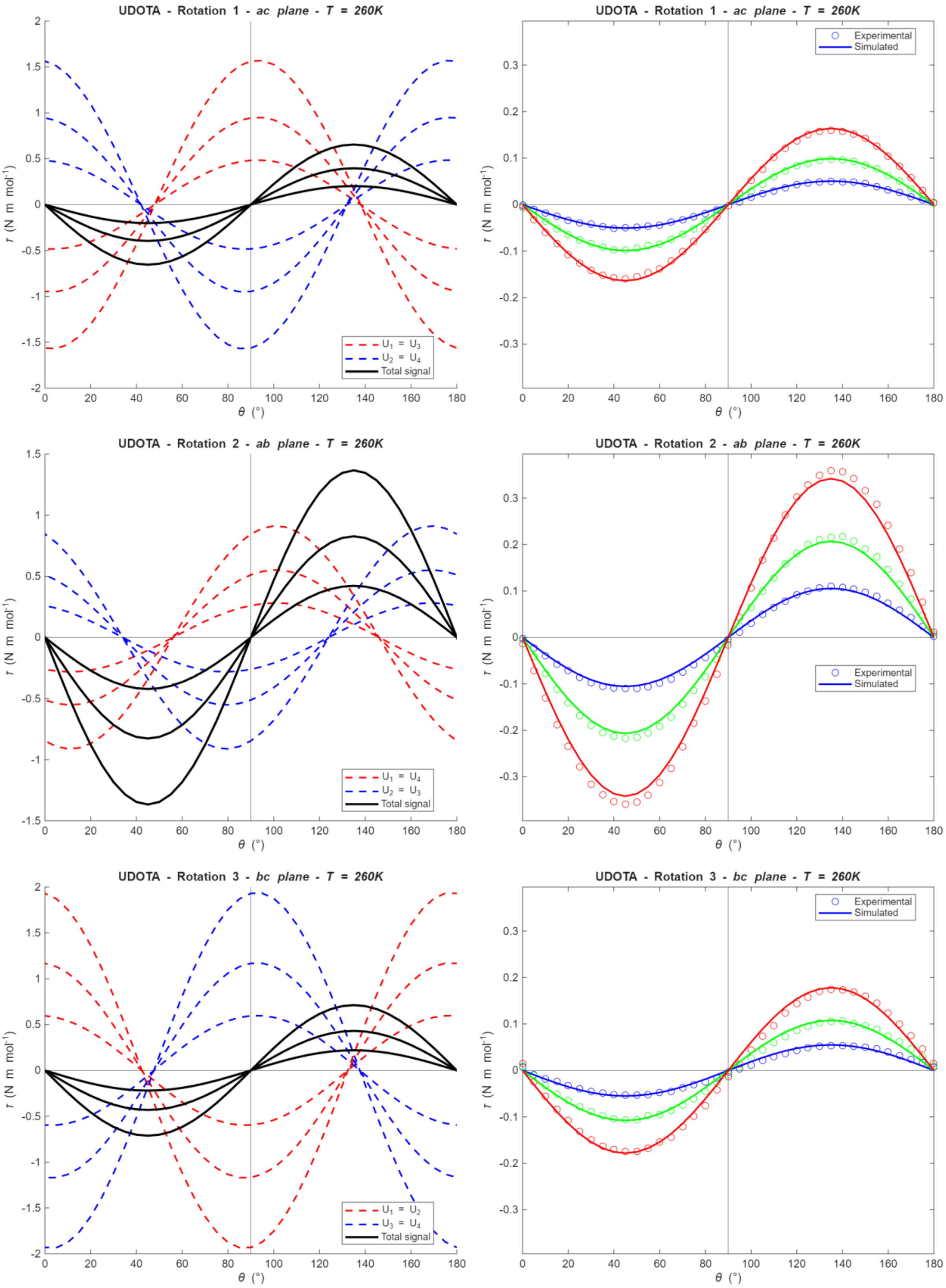

Figure S23 – Left column: contributions of each of the four uranium centres to the total torque signal. Right column: experimental and simulated cantilever torque magnetometry curves obtained at 260K and different magnetic fields: 9T (red), 7T (green) and 5T (blue). Simulations were carried out with parameters obtain from fitting procedure, as discussed in main.

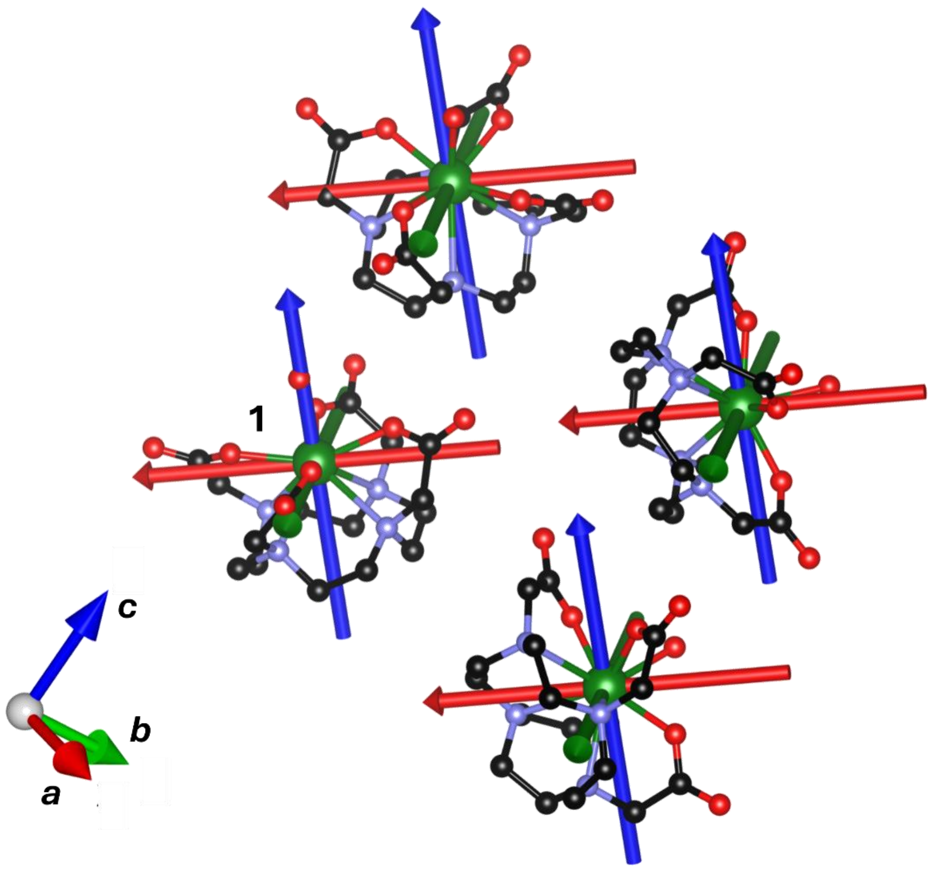

Figure S24. Fitted reference frame superimposed on the four molecules of the unit cell. Color code of the atoms; U, green; C, black, O, red; N, pale blue. Color code of the arrows on the U centers: Red: x, Green: y, Blue: z. The molecule identified as **1** represents the chosen solution, as it is closer to the *ab initio* calculations.

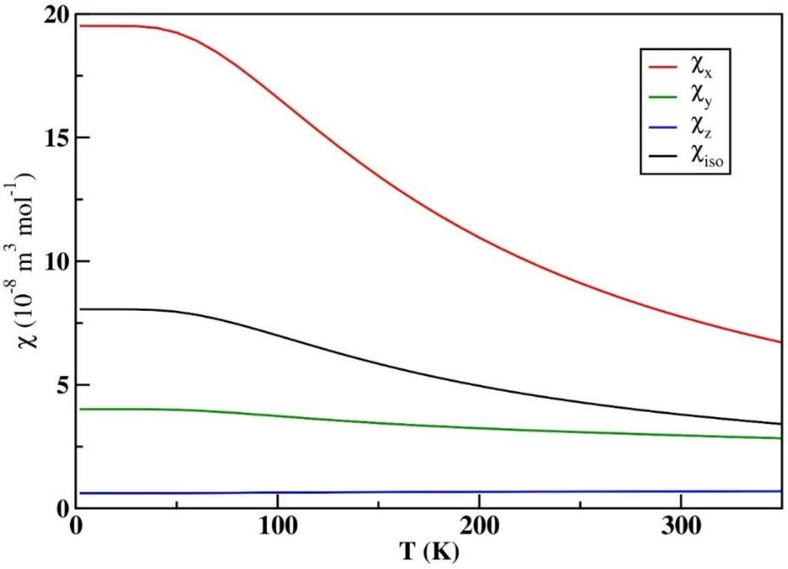

Figure S25 – Magnetic susceptibilities (in 10^{-8} m^3 mol^{-1}) of the **U(DOTA)(H_2O)** complex from SO-MSCASPT2 calculations as a function of temperature. x, y z components of the tensor in red, green and blue, respectively. Powder value in black.

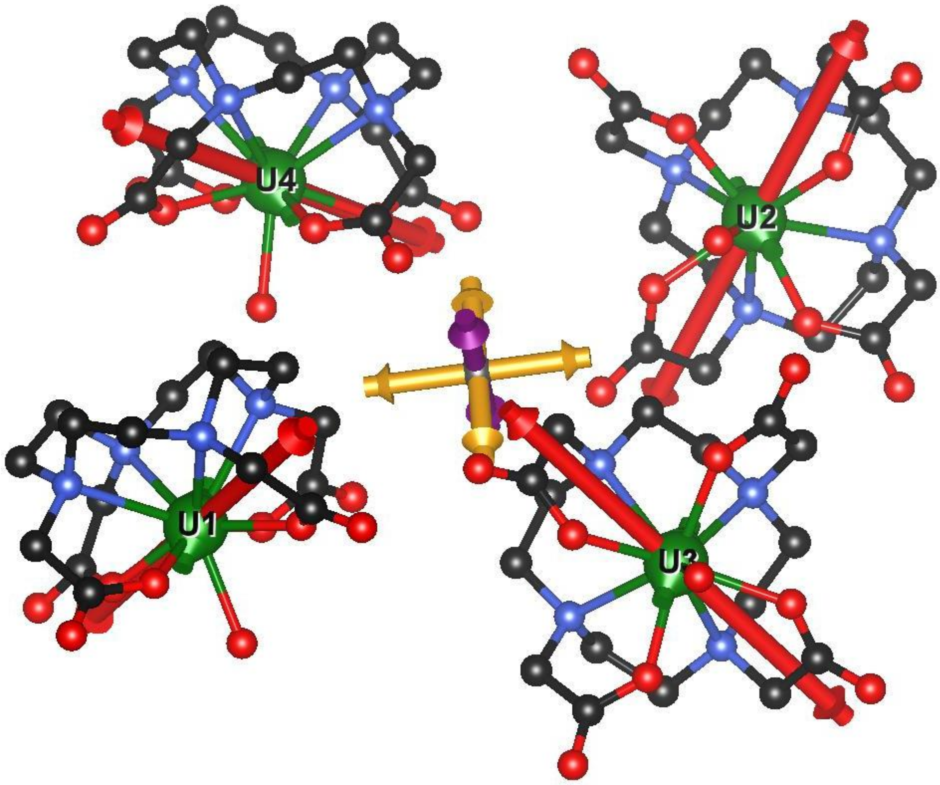

Figure S26 – The four molecules of the unit cell. On each molecule, the arrows show the PAF of the local magnetic susceptibility tensor χ_i^{mol} at 2 K. In the center, the PAF of the crystal magnetic susceptibility tensor χ^{cryst}. Color code of the atoms; U, green; C, black, O, red; N, pale blue. Color code of the arrows on the U centers: x, red; y, green; for the crystal: a, c: orange; b, purple. The length of the arrows is proportional to the corresponding eigenvalue.

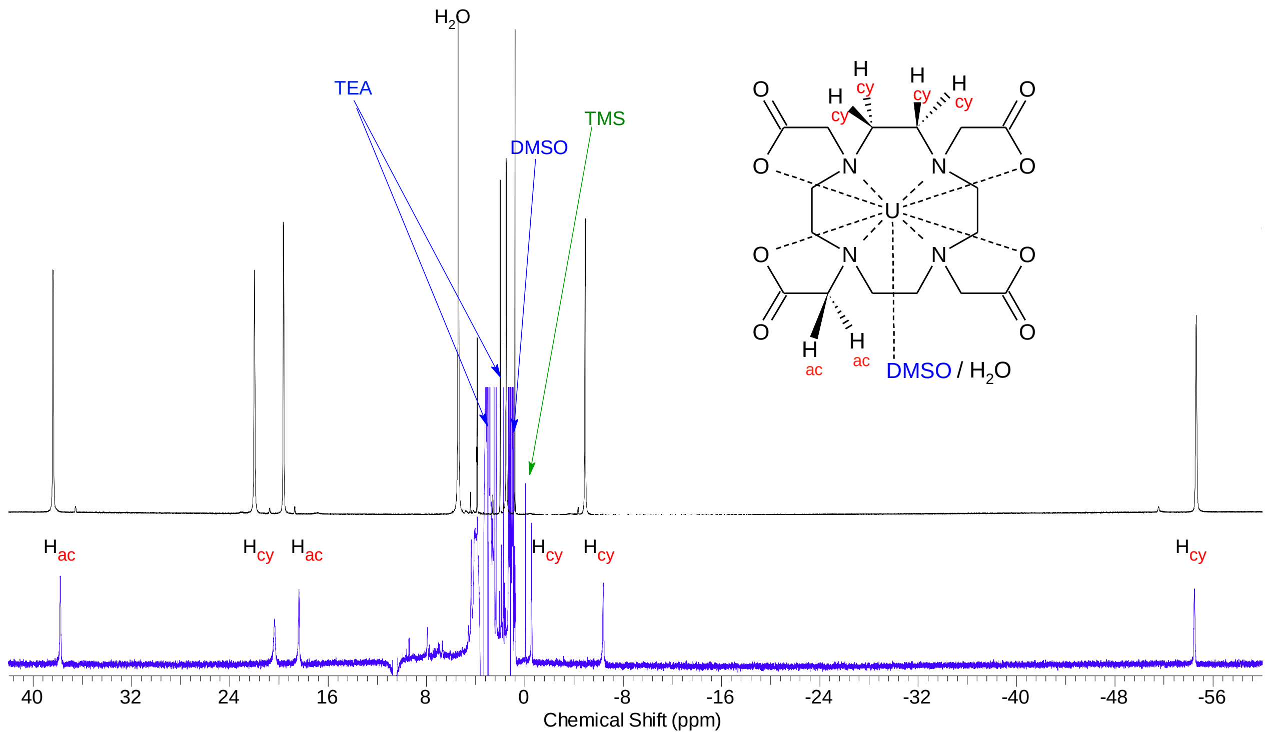

Figure S27: $^1H\{^{13}C\}$ NMR spectra of **U(DOTA)(DMSO)** in DMSO-D6 referenced to internally added TMS ($SiMe_4$) (bottom, blue line) and **U(DOTA)(H_2O)** after THF washes in D_2O (top, black line). Spectra are recorded at room temperature. Chemical shifts of the **U(DOTA)(DMSO)** complex agree with Kent et al. 2019 publication.[1] Spectra were recorded on an Agilent DD2 400MHz spectrometer with a presaturation pulse sequence.

Supplementary Tables

Table S1 – Crystallographic data and refinement details for **U(DOTA)(H_2O)**.

**Compound reference**	**U(DOTA)(H_2O)**
Chemical formula	$C_{16}H_{32}N_4O_{13}U$
Formula mass	726.47
Crystal system	Orthorhombic
a/Å	17.5143(9)
b/Å	8.4627(4)
c/Å	15.9344(8)
α/°	90
β/°	90
γ/°	90
Unit cell volume/$Å^3$	2361.8(2)
Temperature/K	100.0(2)
Space group	$Pca2_1$
No. of formula units/unit cell, *Z*	4
Radiation type	Cu Kα
Absorption coefficient, μ/mm^{-1}	20.002
No. of reflections measured	29435
No. of independent reflections	4142
Data/restraints/parameters	4142/1/303
Final R_1 values (all data)	0.0300
Final $wR_2(F^2)$ values (all data)	0.0660
Final R_1 values ($I > 2\sigma(I)$)	0.0264
Final $wR_2(F^2)$ values ($I > 2\sigma(I)$)	0.0640
Goodness of fit on F^2	1.025
Largest diff. peak and hole ($eÅ^{-3}$)	0.821/-0.717
CCDC number	2478981

Table S2: Crystal-field parameters B_q^k (in cm^{-1}) expressed in Weybourne notation in the symmetric **UDOTA** (**1**), the symmetric **U(DOTA)(H$_2$O)** (**2**), and the XRD **U(DOTA)(H$_2$O)** (**3**) structures. The z-axis is the pseudo C$_4$ axis. For $q \neq 0$, $\bar{B}_q^k = \sqrt{|\,B_q^k|^2 + |\,B_q^{-k}|^2}$

	**1**	**2**	**3**
B_0^2	**-5298**	**-3507**	**-3587**
$\bar{B}_1^2$	0	0	61
$\bar{B}_2^2$	0	230	315
B_0^4	**-138**	**1177**	**1434**
$\bar{B}_1^4$	0	0	113
$\bar{B}_2^4$	0	181	204
$\bar{B}_3^4$	0	0	90
$\bar{B}_4^4$	**3995**	**3982**	**2528**
B_0^6	**-607**	**-134**	**-176**
$\bar{B}_1^6$	0	0	55
$\bar{B}_2^6$	0	335	424
$\bar{B}_3^6$	0	0	88
$\bar{B}_4^6$	**2043**	**2234**	**4137**
$\bar{B}_5^6$	0	0	134
$\bar{B}_6^6$	0	31	138

Table S3 – Euler matrices of the four uranium centres extracted from *ab initio* calculations, as described in main.

U_1	-0.5371	-0.5117	-0.6706
	0.8097	-0.5356	-0.2398
	-0.2365	-0.6718	0.7020
U_2	-0.5371	0.5117	0.6706
	0.8097	0.5356	0.2398
	-0.2365	0.6718	-0.7020
U_3	0.5371	-0.5117	0.6706
	-0.8097	-0.5356	0.2398
	0.2365	-0.6718	-0.7020
U_4	0.5371	0.5117	-0.6706
	-0.8097	0.5356	-0.2398
	0.2365	0.6718	0.7020

Table S4 – Euler matrices of the four uranium centres extracted from fitting procedure of the experimental data, as described in main.

U_1	-0.5582	-0.5231	-0.6440
	0.7970	-0.5539	-0.2409
	-0.2307	-0.6477	0.7261
U_2	-0.5582	0.5231	0.6440
	0.7970	0.5539	0.2409
	-0.2307	0.6477	-0.7261
U_3	0.5582	-0.5231	0.6440
	-0.7970	-0.5539	0.2409
	0.2307	-0.6477	-0.7261
U_4	0.5582	0.5231	-0.6440
	-0.7970	0.5539	-0.2409
	0.2307	0.6477	0.7261

Table S5: Principal components of the magnetic susceptibility tensor (in 10^{-8} m^3 mol^{-1}) of the **U(DOTA)(H_2O)** complex at 2, 150 and 260 K at 9 T obtained from SO-MSCASPT2 calculations (***C***) and fitting procedure (***F***). $\chi_{ax} = \chi_{zz} - 1/2\ (\chi_{yy} + \chi_{xx})$ and $\chi_{rh} = \chi_{xx} - \chi_{yy}$. The error on the fitted values is estimated to be much smaller than the values reported in the table.

**T (K)**	χ_{xx}		χ_{yy}		χ_{zz}		χ_{ax}		χ_{rh}	
	C	***F***	***C***	***F***	***C***	***F***	***C***	***F***	***C***	***F***
2	19.60	19.81	4.01	4.05	0.62	0.62	-11.18	-11.31	15.59	15.76
150	13.46	13.10	3.45	3.26	0.64	0.65	-7.81	-7.53	10.01	9.84
260	8.82	8.66	3.05	2.99	0.68	0.68	-5.25	-5.14	5.77	5.67

Table S6 – SF and SO energy levels (in cm^{-1}) of the **U(DOTA)(H_2O)** complex from SF and SO-MSCASPT2 methods. Ground manifolds (L = 5 for SF, J = 4 for SF) are highlighted in bold.

SF-MSCASPT2		SO-MSCASPT2	
Triplets	Singlets	**0**	8007
**0**	4437	**169**	8079
**722**	4577	**427**	8624
**917**	4584	**879**	8871
**1997**	4718	**1963**	8935
**2291**	5651	**1978**	8971
**2570**	6483	**2056**	9756
**3062**	6735	**2132**	9792
**3407**	7185	**2624**	9823
**3863**	7506	5277	10213
**4222**	9176	5465	10597
**4575**	11290	5620	10642
6173	11341	6115	10738
6250	11665	6210	10785
6367	11849	6729	
6793		6875	
7179		7094	
7698		7334	
7980		7893	

Table S7. Euler matrix of the Einsteinium centre at T = 2 K and B = 5 T. The calculated main components of the susceptibility tensor are: (1909.389531, 6.650115, -3.471813) 10^{-8} m^3 mol^{-1}.

-0.571284	-0.510340	-0.642797
-0.027436	-0.770868	0.636404
-0.820294	0.381203	0.426383

Table S8: SF and SO energy levels (in cm^{-1}) of the **Es(DOTA)(H_2O)** complex from SF and SO-MSCASPT2 methods. Ground manifolds (L=5 for SF, J=15/2 for SO) are highlighted in bold.

SF-MSCASPT2			SO-MSCASPT2	
Sextet	Quartet	Doublet	**0**	7699
**0**	20909	31696	**0**	7699
**99**	21069	31741	**284**	7848
**306**	21129	31795	**284**	7848
**532**	21284	31971	**718**	8076
**1260**	21425	32097	**718**	8076
**1340**	21457	32381	**935**	8161
**1487**	21490	32497	**935**	8161
**1938**	21586	32695	**1166**	8369
**2158**	21600	32893	**1166**	8369
**2353**	21653	32952	**1424**	8670
**2372**	21718	32970	**1424**	8670
6843	21778	33113	**1730**	8936
6897	21820	33208	**1730**	8936
7150	21855	33240	**1841**	
7442			**1841**	
7631				
7678				
7791				

Supplementary Notes

Note 1

The sample, a thin transparent green crystal, was mounted on an acetate foil with two colored faces, using only a minimal amount of grease. The crystal was then indexed to determine the crystallographic axes (*a, b, c*) and to establish their correspondence with the colored sides of the foil. A photograph of the mounted crystal is provided below. The sample (crystal+acetate) was then mounted on the cantilever plate to perform the three rotations described in the main text. The crystal mass was 300(1) µg. Such mass, two orders of magnitude larger than the typical crystal mass used in torque measurements, was here necessary to detect a measurable signal up to 250 K.

Note 2

In the linear regime, the magnetic torque expressed in the orthogonal crystallographic reference frame *abc* of an orthorhombic space group (τ_{abc}) is given by:

$$\vec{\tau}_{abc} = \left[\left(\boldsymbol{\chi_{abc}} \cdot \vec{B}_{abc}\right) \times \vec{B}_{abc}\right]$$

To simplify this expression, we begin by computing the magnetization vector.

$$\vec{M}_{abc} = \boldsymbol{\chi_{abc}} \cdot \vec{B}_{abc} = \begin{bmatrix} \chi_{aa}B_a + \chi_{ab}B_b + \chi_{ac}B_c \\ \chi_{ba}B_a + \chi_{bb}B_b + \chi_{bc}B_c \\ \chi_{ca}B_a + \chi_{cb}B_b + \chi_{cc}B_c \end{bmatrix} = \begin{bmatrix} M_a \\ M_b \\ M_c \end{bmatrix}$$

Then, the torque vector $\vec{\tau}_{abc}$ is given by the cross product:

$$\vec{\tau}_{abc} = \vec{M}_{abc} \times \vec{B}_{abc} = \begin{vmatrix} \hat{e}_a & \hat{e}_b & \hat{e}_c \\ M_a & M_b & M_c \\ B_a & B_b & B_c \end{vmatrix}$$

Expanding this determinant, the components of the torque are:

$$\tau_i = M_j B_k - M_k B_j \qquad with\ i, j, k \in \{a, b, c\}$$

For example, the *a*-component τ_a is given by:

$$\tau_a = M_b B_c - M_c B_b = (\chi_{ba}B_a + \chi_{bb}B_b + \chi_{bc}B_c)B_c - (\chi_{ca}B_a + \chi_{cb}B_b + \chi_{cc}B_c)B_b$$

Considering that the magnetic susceptibility tensor $\boldsymbol{\chi_{abc}}$ is diagonal in the crystallographic frame, i.e. $\chi_{ij} = 0$ for $i \neq j$, the off-diagonal terms vanish, yielding:

$$\tau_a = \chi_{bb} B_b B_c - \chi_{cc} B_b B_c = \Delta\chi_{bc} B_b B_c$$

Similarly, the other components are:

$$\tau_b = \chi_{cc} B_a B_c - \chi_{aa} B_a B_c = \Delta\chi_{ca} B_a B_c$$

$$\tau_c = \chi_{aa} B_a B_b - \chi_{bb} B_a B_b = \Delta\chi_{ab} B_a B_b$$

Therefore,

$$\vec{\tau}_{abc} = \begin{bmatrix} \Delta\chi_{bc} B_b B_c \\ \Delta\chi_{ca} B_a B_c \\ \Delta\chi_{ab} B_a B_b \end{bmatrix}$$

To obtain the scalar torque projected along the rotation axis $\vec{r}_{abc}$, we compute the dot product:

$$\tau = \vec{\tau}_{abc} \cdot \vec{r}_{abc} = \Delta\chi_{bc} B_b B_c r_a + \Delta\chi_{ca} B_a B_c r_b + \Delta\chi_{ab} B_a B_b r_c$$

This expression can be compactly written using the Levi-Civita symbol ε_{ijk} as:

$$\tau_i = \frac{1}{2} \sum_{j,k} \varepsilon_{ijk} \Delta\chi_{jk} B_j B_k r_i \quad with\ i, j, k \in \{a, b, c\}$$

Here, the factor 1/2 accounts for the double counting over the indices j and k.

Note 3

U(IV) is a non-Kramers ion. Without any spatial symmetry, none of its states are degenerate, and none of the states is magnetic. The energies are given in Table S5. The nine lowest states arise from the ground *J* = 4 manifold of the free ion. The crystal-field parameters (CFPs) expressed in Wybourne notation are given in Table S2. The symmetrized complex shows a C4 symmetry and only B_0^2, B_0^4, B_0^6, $\bar{B}_4^4$ and $\bar{B}_4^6$ are non-zero. B_0^2 is by far the largest diagonal CFP and is negative. Since U(IV) has 2 *f* electrons, α_J (reduced matrix element) is negative and the ground state corresponds to an almost pure $M_J = 0$ component (with the C4 axis as the z hard axis)[2]. The other $|M_J|$ states follow an increasing energy ladder (*i.e.* $|M_J| = 4$ is the highest state). The two states with $|M_J| = 2$ are largely split by the $\bar{B}_4^k$ (*k* = 4, 6) parameters. The effect of the apical water molecule is a descent in symmetry to

C2, with the resulting $\bar{B}_2^k$ being nonzero. Finally, the molecule gets distorted due to the crystal packing (C1 symmetry). The two first excited states, $|1\rangle$ and $|2\rangle$, with $|M_J| = 1$ are, in this condition, split by ≈ 250 cm^{-1}.

The eigenvalues of the magnetic susceptibility tensor are given in Table S6. The Principal Axes Frame is almost independent of temperature, with *x* and *y* pointing towards the bonding carboxylate oxygen atoms. χ_x and χ_y are very different at low temperature ($19\cdot10^{-8}$ and $4\cdot10^{-8}$ m^3mol^{-1}, respectively). There is no clear structural difference between the x and y directions, the U-O distances are very similar, with very small distortions. The hard axis points towards the apical water molecule. The temperature dependence is shown in Figure S25. Up to 50 K, the values are constant, and then, χ_x decreases strongly while χ_y decreases smoothly. The isotropic value of $4\cdot10^{-8}$ m^3mol^{-1} at room temperature is within the usual range for U(IV) complexes (for example, $5\cdot10^{-8}$ for $[U(DPA)_3]^{2-}$ and $3.6\cdot10^{-8}$ m^3mol^{-1} for $[U(NO3)_6]$.[3,4]

From this picture, one can extract information about the crystal anisotropy tensor. As reported in Figure S3, until 50 K the crystallographic $\boldsymbol{\chi}^{\boldsymbol{cryst}}$ is planar, with identical values for *a* and *c*. As shown in Figure S26, this is due to an arrangement of the four molecules of the unit cell such that the projection of the four molecular $\boldsymbol{\chi}_{\boldsymbol{i}}^{\boldsymbol{mol}}$ in the *a* and *c* axes is identical at low temperature. From 100 K, the population of the first excited state changes the values of the molecular tensor, and the projections in the *a* and *c* axes differ.

Note 4

All reagents were purchased from commercial suppliers and used as received. H_4DOTA (75 mg, 0.19 mmol, purchased from CheMatech-mdt.com) were dissolved in 10 mL of anhydrous dimethyl sulfoxide (DMSO) containing 404mg (75 mmol) of triethylamine (TEA). 200 mL of uranyl nitrate solution (155 g/L in 2M HNO_3) available in the lab (0.13 mmol of U^{VI}) were placed in 1mL of HNO_3 2M and reduced with an excess of Rongalite (150 mg, 10 eq). The reduction in U^{IV} was checked by UV-vis spectroscopy (absorption at 650 nm of U^{IV} and no band in between 390-450nm of U^{VI}) and precipitated with a large excess of NaOH (one pellet ≈100 mg, 2.5 mmol). The solid was washed 3 times with distilled water, recovered with 500 mL of concentrated HCl (37%), and dried under nitrogen flow overnight. The resulting solid (UCl_4) was dissolved in the DMSO solution containing neutralized DOTA ligand (DOTA(TEA)$_4$) and placed at 60°C overnight.

The formation of **U(DOTA)(DMSO)** was monitored by ^{1}H NMR spectroscopy (FigureS26, bottom). After completion of the reaction, the complex was precipitated by dropwise addition of the DMSO solution in a $CHCl_3$ phase (10 mL). The resulting powder was washed three times with tetrahydrofuran (THF, 400 mL each time) then dried at room temperature under nitrogen flow.

The DMSO replacement by H_2O in apical position was carried out by dissolving **U(DOTA)(DMSO)** in a minimum amount of water (200 mL) and precipitated by addition of 1mL THF. To ensure DMSO removal, this operation was repeated several times. The final light green solid compound was washed twice with THF (until THF was colourless) and then dried at room temperature under nitrogen flow. The formation of **U(DOTA)(H_2O)** was confirmed by ^{1}H NMR spectroscopy (Figure S27, top). 400 mL of a light green aqueous solution was obtained. The uranium concentration measured by ICP-MS spectrometry resulting in 25.1 mmol/L of **U(DOTA)(H_2O)**. The overall yield is approximately 8% (due to the various washing procedures). To obtain single crystals of the product, the light green solid obtained was redissolved in a minimal amount of water and repeatedly washed with THF. The biphasic mixture was centrifuged for 3 minutes at 3000 rpm, after which the upper THF phase was discarded. This washing–centrifugation cycle was repeated until the THF phase became completely colourless. Slow evaporation of the aqueous solution led to the deposition of crystals.

References

(1) Kent, G. T.; Wu, G.; Hayton, T. W. Synthesis and Crystallographic Characterization of the Tetravalent Actinide-DOTA Complexes [An IV (κ 8 -DOTA)(DMSO)] (An = Th, U). *Inorg. Chem.* **2019**, *58* (13), 8253–8256. https://doi.org/10.1021/acs.inorgchem.9b00736.

(2) Alessandri, R.; Zulfikri, H.; Autschbach, J.; Bolvin, H. Crystal Field in Rare-Earth Complexes: From Electrostatics to Bonding. *Chem. – A Eur. J.* **2018**, *24* (21), 5538–5550. https://doi.org/10.1002/chem.201705748.

(3) Islam, M. A.; Autillo, M.; Guérin, L.; Tamain, C.; Moisy, P.; Bolvin, H.; Berthon, C. Dipolar and Contact Paramagnetic NMR Chemical Shifts in An IV Complexes with Dipicolinic Acid Derivatives. *Inorg. Chem.* **2022**, *61* (27), 10329–10341. https://doi.org/10.1021/acs.inorgchem.2c00845.

(4) Autillo, M.; Illy, M.-C.; Briscese, L.; Islam, M. A.; Bolvin, H.; Berthon, C. Paramagnetic Properties of [An IV (NO 3) 6] 2− Complexes (An = U, Np, Pu) Probed by NMR Spectroscopy and Quantum Chemical Calculations. *Inorg. Chem.* **2024**, *63* (28), 12969–12980. https://doi.org/10.1021/acs.inorgchem.4c01694.
